# Mechanics of Small-Scale Spherical Inclusions Using Nonlocal Poroelasticity Integrated with Light Gradient Boosting Machine

**DOI:** 10.3390/mi15020210

**Published:** 2024-01-30

**Authors:** Ali Farajpour, Wendy V. Ingman

**Affiliations:** 1 Adelaide Medical School, University of Adelaide, The Queen Elizabeth Hospital, Woodville South, SA 5011, Australia; wendy.ingman@adelaide.edu.au; 2 Robinson Research Institute, University of Adelaide, Adelaide, SA 5006, Australia

**Keywords:** nonlocal continuum mechanics, scale effects, inclusions, light gradient boosting machine, poroelasticity

## Abstract

Detecting inclusions in materials at small scales is of high importance to ensure the quality, structural integrity and performance efficiency of microelectromechanical machines and products. Ultrasound waves are commonly used as a non-destructive method to find inclusions or structural flaws in a material. Mathematical continuum models can be used to enable ultrasound techniques to provide quantitative information about the change in the mechanical properties due to the presence of inclusions. In this paper, a nonlocal size-dependent poroelasticity model integrated with machine learning is developed for the description of the mechanical behaviour of spherical inclusions under uniform radial compression. The scale effects on fluid pressure and radial displacement are captured using Eringen’s theory of nonlocality. The conservation of mass law is utilised for both the solid matrix and fluid content of the poroelastic material to derive the storage equation. The governing differential equations are derived by decoupling the equilibrium equation and effective stress–strain relations in the spherical coordinate system. An accurate numerical solution is obtained using the Galerkin discretisation technique and a precise integration method. A Dormand–Prince solution is also developed for comparison purposes. A light gradient boosting machine learning model in conjunction with the nonlocal model is used to extract the pattern of changes in the mechanical response of the poroelastic inclusion. The optimised hyperparameters are calculated by a grid search cross validation. The modelling estimation power is enhanced by considering nonlocal effects and applying machine learning processes, facilitating the detection of ultrasmall inclusions within a poroelastic medium at micro/nanoscales.

## 1. Introduction

Accurate theoretical models of background–inclusion media are essential in the quantitative determination of mechanical properties of poroelastic materials such as biological tissues, hydrogels, micro-fibrous scaffolds and micro-porous polymers, in which inclusions such as tumours and structural anomalies are common [1]. These theoretical models allow us to precisely evaluate mechanical features and compare them on an elastography image (elastogram) in imaging techniques like ultrasound elastography [2]. A number of mathematical models have been developed for the mechanical behaviour of poroelastic materials with inclusions in recent years. Shin et al. [3] used Eshelby’s model of elasticity, an analytical method of estimating the elastic properties of inclusion–matrix media, in order to determine strain and stress components inside breast tissues with ellipsoidal lesions as inclusions. In another study, Goswami and his co-workers [4] developed a theoretical platform using the Caley–Hamilton theorem and classical elasticity to analyse the shear induced nonlinear mechanics in phantoms with undesirable inclusions under finite deformations. A poroelastic model was also presented by Islam and Righetti [5] using the conventional poroelasticity theory for investigating the mechanics of biological tissues containing spherical tumours. Moreover, a numerical mathematical approach was proposed to study large-scale elastic bodies with thin inclusions based on a scale-free elasticity theory and finite element technique [6]. Costa and Gentile [7] developed a discrete doublet model of mechanics to simulate ultrasound wave propagation within biological tissues as the poroelastic material, excluding any potential inclusions. In a recent study conducted by Favata et al. [8], it has been shown that the mechanical behaviour of biological inclusions at microscale levels is different from those at large scales. The development of microscale premalignant inclusions leads to stiffness softening, while the presence of large-scale inclusions is associated with a hardening behaviour, known as the soft-cell solid tumour paradox [9]. More recently, mathematical models of poroelasticity [10] and an artificial neural network technique [11] have been developed to capture Poisson’s ratio in abnormalities and estimate mechanical stiffness in inhomogeneous materials.

However, at nano and microscales, the dimensionless mechanical characteristics of a substance are highly sensitive to its size [12,13,14,15]. This widely reported phenomenon is known as scale (size) dependency [16,17] and is associated with several underlying factors including molecular interactions [18] and stiffness alteration [19]. As classical local elasticity and poroelasticity models are formulated on the basis of scale-free theories of continuum mechanics, they lack the ability to capture size effects and, thus, fail to accurately estimate the mechanical response at nano and microscales. Peddieson et al. [20] applied a version of nonlocal elasticity, which was first introduced by Eringen [21], to develop scale-dependent beam models suitable for describing the mechanics of nanoscale devices such as small-scale actuators with nanocantilevers as building blocks. Following this pioneering work, several researchers across the world have extended the application of nonlocal theories to other small-scale structures and devices such as nanobeams [22], nanoscale sensors [23], nanoplates [24,25,26] and fluid-conveying microtubes [27,28].

In addition to fundamental small-scale solid structures, modified nonlocal models have been utilised to assess and predict the mechanical behaviour of poroelastic, viscoelastic and biological materials of small sizes [29,30,31]. These structures include, but are not limited to, microtubules [32,33], nanoporous materials with surface effects [34] and lipid micro-tubules [35]. In all of these valuable studies, it has been demonstrated that scale effects have a vital role to play in mechanical deformation. Stress nonlocality described by the Eringen nonlocal elasticity (nonlocal scale effect) is associated with small-scale interactions, leading to substantial stiffness reduction in the structure. Furthermore, it has been recently demonstrated that nonlocal models hold great promise as highly accurate mathematical tools for the description and design of microscale systems and phenomena, especially in biology such as microscale migration of cells [36], microelectromechanical response [37] and wave propagation in biological tissues [38].

To the best of our knowledge, to date, no nonlocal scale-dependent poroelastic model has been developed for the mechanical deformation of materials with small-scale inclusions. In imaging technologies such as ultrasound and optical elastography that utilise the mechanical properties of a given poroelastic material to detect abnormalities, mathematical models play a crucial role in the accurate visualisation of mechanical characteristics [5,39]. However, conventional mathematical models are formed based on classical elasticity theories that fail to capture size effects and thus cannot be employed at ultrasmall levels [13]. In this paper, stress nonlocality-based size effects on the mechanical response of poroelastic materials with small-scale inclusions are studied for the first time. Furthermore, this research represents the first integration of nonlocal elasticity and a light gradient boosting machine for addressing inclusion problems. The proposed nonlocal scale-dependent model of poroelasticity developed in this paper could be used in elastography imaging techniques to accurately detect inclusions of ultrasmall sizes.

A case study of a potential application of this model is investigated. The detection of small-scale tumours in breast tissue (poroelastic medium) is considered as the undesirable microscale inclusion. To include size dependency, nonlocal elasticity theory is utilised. The influences of tissue fluid content, hydraulic conductivity and microfiltration are captured by using a modified version of poroelasticity theory. The governing differential equations are derived by decoupling the scale-dependent constitutive relations and equilibrium equation in the spherical coordinate system. To discretise the decoupled differential equations, Galerkin method is employed. An accurate solution is presented with the use of the precise integration method and Dormand–Prince technique. A light gradient boosting machine learning model is also presented to extract and learn the underlying patterns in the mechanical behaviour of spherical poroelastic inclusions. To optimise the model, a grid search cross validation approach is implemented. A detailed examination of the effects of nonlocal scale coefficient and inclusion size on the time-dependent fluid pressure and radial displacement is presented.

## 2. Nonlocal Poroelasticity Modelling

In this section, a nonlocal scale-dependent model is developed for poroelastic materials including small-scale spherical inclusions. In biomedical applications, small-scale inclusions of interest to be detected by ultrasound imaging or other imaging techniques are usually a clump of cancer cells with a stiffness lower than healthy cells [8,9]. This softening behaviour can be effectively incorporated using nonlocal continuum mechanics as stress nonlocality is associated with structural stiffness softening. An appropriate model for this case is a refined combination of Eringen’s nonlocal theory and poroelasticity to account for both stiffness softening and fluid effects.

The conservation of mass for the fluid content of a given poroelastic material can be written as [40]

(1) $$\nabla \cdot \left(\rho_{f}nV_{f}\right)+\frac{\partial }{\partial t}\left(\rho_{f}n\right)=0,$$

where $\rho_{f}$, $V_{f}$ and *n* are the fluid density, fluid velocity and porosity, respectively. Moreover, ∇ , “.” and *t* indicate the gradient operator, dot product and time, respectively. Similarly, the mass balance equation for the solid matrix is obtained as

(2) $$\nabla \cdot \left\{\rho_{s}\left(1-n\right)V_{s}\right\}+\frac{\partial }{\partial t}\left\{\rho_{s}\left(1-n\right)\right\}=0.$$

Here $\rho_{s}$ and $V_{s}$ are the solid matrix density and velocity, respectively. Assuming that the fluid part and solid components (particles) are not compressible, and combining the mass balance Equations (1) and (2), one obtains

(3) $$\nabla \cdot d_{sp}+\nabla \cdot V_{s}=0,$$

where $d_{sp}$ is the specific discharge that is associated with the relative velocity ($V_{rel}$) as

(4) $$d_{sp}=nV_{rel},$$

where

(5) $$V_{rel}=V_{f}-V_{s}.$$

It is assumed that the fundamental solid particles and fluid part of the poroelastic material are individually incompressible [40]. However, the relative sliding, rotating and movement between these components allow for the overall material and the solid phase as a whole to exhibit compressibility [5]. The volumetric strain of the whole solid part (ε) is related to the displacement vector ($u_{s}$) by

(6) $$\varepsilon =\nabla \cdot u_{s}.$$

Using Equations (3) and (6), the following relation is obtained

(7) $$\nabla \cdot d_{sp}+\frac{\partial \varepsilon }{\partial t}=0.$$

According to Darcy’s law, the specific discharge of a porous material is proportionally dependent on the fluid pressure gradient (∇p) and the gravity vector (g) as [40]

(8) $$d_{sp}=-\frac{\eta_{pm}}{\mu_{f}}\left(\nabla p-\rho_{f}g\right),$$

in which $\eta_{pm}$, $\mu_{f}$ and p represent the material permeability, fluid viscosity and fluid pressure, respectively. Substituting Equation (8) into Equation (7) and considering the effect of potential microfiltration [41], the final version of the mass balance equation (storage equation) is obtained as

(9) $$\frac{\partial \varepsilon }{\partial t}+\chi_{tot}p=\frac{\lambda_{hc}}{\gamma_{vw}}\nabla^{2}p,$$

where $\lambda_{hc}$ and $\gamma_{vw}$ denote the hydraulic conductivity and volumetric weight of the fluid, which are defined by $\lambda_{hc}=\eta_{pm}\gamma_{vw}/\mu_{f}$ and $\gamma_{vw}=g\rho_{f}$, respectively. $\nabla^{2}$ is the Laplace operator. In the case of biological inclusions such as solid tumours, $\chi_{tot}$ is the total microfiltration coefficient, which is expressed by [5,41]

(10) $$\chi_{tot}=\chi_{vas}+\chi_{lym},$$

where

(11) $$\begin{array}{l} \chi_{vas}=\frac{k_{vas}S_{vas}}{V_{vas}},\\ \chi_{lym}=\frac{k_{lym}S_{lym}}{V_{lym}}, \end{array}$$

in which $\chi_{lym}$ and $\chi_{vas}$ indicate the tumour lymphatic and vascular microfiltration coefficients, respectively. $k_{vas}$, $S_{vas}$ and $V_{vas}$ stand for the vascular permeability, surface area and volume, respectively. Similarly, $k_{lym}$, $S_{lym}$ and $V_{lym}$ are the lymphatic permeability, surface area and volume, respectively. Equation (9) represents the storage equation of poroelastic materials from biological tissues to porous micro-polymers. For applications in which there is no microfiltration effect, $\chi_{tot}$ is set to zero.

For spherical poroelastic inclusions, the components of the total stress tensor ($\sigma_{ij}$) are related to the effective stress (${\sigma^{\prime }}_{ij}$) and fluid pressure (*p*) as

(12) $$\begin{array}{l} \sigma_{rr}={\sigma^{\prime }}_{rr}+p,\\ \sigma_{\theta \theta }={\sigma^{\prime }}_{\theta \theta }+p,\\ \sigma_{\phi \phi }={\sigma^{\prime }}_{\phi \phi }+p,\\ \sigma_{r\theta }={\sigma^{\prime }}_{r\theta },\\ \sigma_{r\phi }={\sigma^{\prime }}_{r\phi },\\ \sigma_{\theta \phi }={\sigma^{\prime }}_{\theta \phi }. \end{array}$$

**Figure 1:**
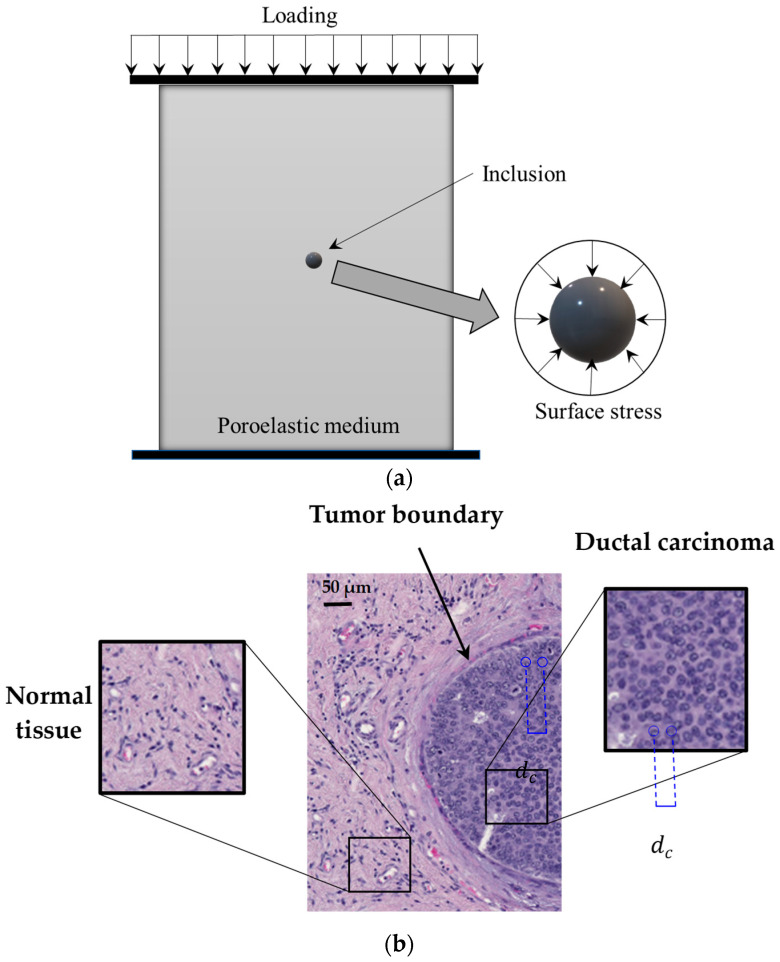
(**a**) Schematic representation of a poroelastic material with a spherical inclusion under compressive loading. (**b**) A microscopic image of an early human breast tumour in the form of carcinoma in situ as a small-scale biological inclusion (haematoxylin and eosin stains of tissue sections [43]). The internal length-scale parameter could be related to the average distance between individual cells ($d_{c}$). Within the inclusion, the average distance between individual cells is much less than that of the healthy background tissue [43].

Effective stress components can be interpreted as the parts of the total stress tensor that are responsible for porous material deformation. To detect an inclusion in a given poroelastic medium, in many practical cases, it is assumed that the average size of the inclusion is very small compared to the medium size as the whole size [5,42]. Figure 1 shows the schematic representation of a poroelastic medium including a small-scale inclusion of a spherical shape. A slight compressive load is applied on the top surface of the medium. A compressor plate is used to make sure that the compressive load is uniformly distributed on the medium surface. In practical applications, the compressive force is commonly applied by utilising an ultrasound transducer, and a number of force sensors can be used to measure the magnitude of the loading. Since the inclusion size is very small compared to the distance from the inclusion centre to the loading location, it is reasonable to assume that the spherical inclusion is subject to a symmetric uniform radial load, as indicated in Figure 1. Therefore, the normal stress along the θ direction is the same as that of the ϕ direction ($\sigma_{\theta \theta }=\sigma_{\phi \phi }$). The equilibrium differential equation is given by

(13) $$\frac{\partial \sigma_{rr}}{\partial r}+\frac{2\left(\sigma_{rr}-\sigma_{\theta \theta }\right)}{r}=0,$$

where *r* denotes the radial distance from the inclusion centre. Substituting Equation (12) into the above equilibrium equation leads to

(14) $$\frac{\partial {\sigma^{\prime }}_{rr}}{\partial r}+\frac{2\left({\sigma^{\prime }}_{rr}-{\sigma^{\prime }}_{\theta \theta }\right)}{r}+\frac{\partial p}{\partial r}=0,$$

The average size of the inclusion is very small compared to the background medium, and thus we can assume that the loading condition is spherically symmetric on the inclusion surface [5]; this assumption is made as this study deals with the scale-dependent mechanics of ultrasmall inclusions. Furthermore, it is assumed that the deflection caused by external loading is small, leading to geometrical linearity assumption for strain-displacement relations. In practical applications, especially in biomedical scenarios, gentle mechanical forces are applied using devices such as an ultrasound transducer or a mechanical probe. These loading systems are designed to be comfortable and painless and induce only slight loads on patients’ bodies, consequently resulting in small displacements and geometric linearity.

To capture the scale effects that are related to the effective stress nonlocality, Eringen’s theory is used [44]. According to this theory, the effective stress at a particular point depends not only on the strain components at that point but also on the strain components at all other points of the porous material. The stress nonlocality assumption made in Eringen’s theory allows us to take into account small-scale interactions from a mechanical point of view. Based on the nonlocal theory of poroelasticity, the effective stresses are expressed in terms of strain components as

(15) $$\left[1-{\left(e_{0}a_{c}\right)}^{2}\nabla^{2}\right]{\sigma^{\prime }}_{rr}=-\left(2\mu \varepsilon_{rr}+\lambda \varepsilon \right)=-\left[2G\varepsilon_{rr}+\left(K-\frac{2}{3}G\right)\varepsilon \right],$$

(16) $$\left[1-{\left(e_{0}a_{c}\right)}^{2}\nabla^{2}\right]{\sigma^{\prime }}_{\theta \theta }=-\left(2\mu \varepsilon_{\theta \theta }+\lambda \varepsilon \right)=-\left[2G\varepsilon_{\theta \theta }+\left(K-\frac{2}{3}G\right)\varepsilon \right],$$

where

(17) $$\nabla^{2}\left(•\right)=\frac{\partial^{2}}{\partial r^{2}}\left(•\right)+\frac{2}{r}\frac{\partial }{\partial r}\left(•\right)=\frac{1}{r}\frac{\partial^{2}\left(r\left(•\right)\right)}{\partial r^{2}}.$$

Here μ and λ are Lamé coefficients, and *G* and *K* represent the shear and bulk moduli of the spherical inclusion, respectively. $e_{0}$ and $a_{c}$ are a calibration parameter and an internal characteristics size, respectively. The product of these two features is widely known as the nonlocal parameter ($e_{0}a_{c}$). In addition, $\varepsilon_{ij}$ and ε are the strain component and volumetric strain, respectively. This internal length-scale parameter could be associated with the average distance between fundamental components within the inclusion. Figure 1b gives an example of a biomedical inclusion in the form of early breast tumours. In the tumour, individual cells have developed in closer proximity to each other compared to the surrounding healthy tissue. The conditions of the above nonlocal constitutive equations are stress–strain linearity and material homogeneity. Furthermore, reduced partial differential equations of nonlocal elasticity, which were introduced by Eringen [44] for a group of physically admissible kernels, have been utilised. These constitutive equations were obtained from the integral form of nonlocal elasticity by assuming that the nonlocal modulus is Green’s function of a linear differential operator [44].

For spherical inclusions, strain components can be written as

(18) $$\varepsilon_{rr}=\frac{\partial u_{r}}{\partial r},\varepsilon_{\theta \theta }=\varepsilon_{\phi \phi }=\frac{u_{r}}{r},\varepsilon_{r\theta }=\varepsilon_{\theta \phi }=\varepsilon_{r\phi }=0,$$

(19) $$\varepsilon =\varepsilon_{rr}+\varepsilon_{\theta \theta }+\varepsilon_{\phi \phi }=\frac{\partial u_{r}}{\partial r}+\frac{2u_{r}}{r}=\frac{1}{r^{2}}\frac{\partial }{\partial r}\left(r^{2}u_{r}\right).$$

In Equations (18) and (19), $u_{r}$ is the displacement along the radial direction. The effective stress–strain Equations (15) and (16), together with the equilibrium Equation (14), form three coupled partial differential equations that govern the deformation behaviour of the inclusion. To calculate the displacement, strain and fluid pressure, these differential equations need to be decoupled first. For the sake of brevity, the procedure of decoupling is not mentioned here. Substituting Equations (17)–(19) into the resultant decoupled equation leads to

(20) $$\begin{array}{l} \lambda \left(\frac{\partial^{2}u_{r}}{\partial r^{2}}+\frac{2}{r}\frac{\partial u_{r}}{\partial r}-\frac{2}{r^{2}}u_{r}\right)+2\mu \frac{\partial^{2}u_{r}}{\partial r^{2}}\\ +\frac{4\mu }{r}\left(\frac{\partial u_{r}}{\partial r}-\frac{u_{r}}{r}\right)-\left[\frac{\partial p}{\partial r}-{\left(e_{0}a_{c}\right)}^{2}\left(\frac{\partial^{3}p}{\partial r^{3}}+\frac{2}{r}\frac{\partial^{2}p}{\partial r^{2}}\right)\right]\\ -\frac{1}{r^{2}}{\left(e_{0}a_{c}\right)}^{2}\left\{2\left(\lambda +2\mu \right)r\left(\frac{\partial^{3}u_{r}}{\partial r^{3}}+\frac{4}{r}\frac{\partial^{2}u_{r}}{\partial r^{2}}\right)\right.\\ +\left(\lambda +2\mu \right)r^{2}\left(\frac{\partial^{4}u_{r}}{\partial r^{4}}+\frac{4}{r}\frac{\partial^{3}u_{r}}{\partial r^{3}}-\frac{4}{r^{2}}\frac{\partial^{2}u_{r}}{\partial r^{2}}\right)\\ -2\left[\frac{\partial p}{\partial r}-{\left(e_{0}a_{c}\right)}^{2}\left(\frac{\partial^{3}p}{\partial r^{3}}+\frac{2}{r}\frac{\partial^{2}p}{\partial r^{2}}\right)\right]\\ -4r\left[\frac{\partial^{2}p}{\partial r^{2}}-{\left(e_{0}a_{c}\right)}^{2}\left(\frac{\partial^{4}p}{\partial r^{4}}+\frac{2}{r}\frac{\partial^{3}p}{\partial r^{3}}-\frac{2}{r^{2}}\frac{\partial^{2}p}{\partial r^{2}}\right)\right]\\ \left.-r^{2}\left[\frac{\partial^{3}p}{\partial r^{3}}-{\left(e_{0}a_{c}\right)}^{2}\left(\frac{\partial^{5}p}{\partial r^{5}}+\frac{2}{r}\frac{\partial^{4}p}{\partial r^{4}}-\frac{4}{r^{2}}\frac{\partial^{3}p}{\partial r^{3}}+\frac{4}{r^{3}}\frac{\partial^{2}p}{\partial r^{2}}\right)\right]\right\}\\ -\frac{2}{r^{3}}{\left(e_{0}a_{c}\right)}^{2}\left\{\left(\lambda +2\mu \right)r^{2}\left(\frac{\partial^{3}u_{r}}{\partial r^{3}}+\frac{4}{r}\frac{\partial^{2}u_{r}}{\partial r^{2}}\right)\right.\\ -2r\left[\frac{\partial p}{\partial r}-{\left(e_{0}a_{c}\right)}^{2}\left(\frac{\partial^{3}p}{\partial r^{3}}+\frac{2}{r}\frac{\partial^{2}p}{\partial r^{2}}\right)\right]\\ \left.-r^{2}\left[\frac{\partial^{2}p}{\partial r^{2}}-{\left(e_{0}a_{c}\right)}^{2}\left(\frac{\partial^{4}p}{\partial r^{4}}+\frac{2}{r}\frac{\partial^{3}p}{\partial r^{3}}-\frac{2}{r^{2}}\frac{\partial^{2}p}{\partial r^{2}}\right)\right]\right\}\\ +\frac{2}{r^{2}}{\left(e_{0}a_{c}\right)}^{2}\left\{4\mu \frac{\partial^{2}u_{r}}{\partial r^{2}}-\left[\frac{\partial p}{\partial r}-{\left(e_{0}a_{c}\right)}^{2}\left(\frac{\partial^{3}p}{\partial r^{3}}+\frac{2}{r}\frac{\partial^{2}p}{\partial r^{2}}\right)\right]\right\}=0. \end{array}$$

Using the relation of the volumetric strain given by Equation (19), the mass balance Equation (9) can be rewritten as

(21) $$\frac{\partial }{\partial t}\left(\frac{\partial u_{r}}{\partial r}+\frac{2u_{r}}{r}\right)+\chi_{tot}p=\frac{\lambda_{hc}}{\gamma_{vw}}\left(\frac{\partial^{2}p}{\partial r^{2}}+\frac{2}{r}\frac{\partial p}{\partial r}\right).$$

From the above equations, it is found that the equilibrium equation in terms of the radial displacement and pressure is dependent on nonlocal influences, while the mass balance equation is not affected by the stress nonlocality, as expected. When the scale effects associated with the stress nonlocality are ignored (i.e., $e_{0}a_{c}=0$), the governing differential equations of the poroelastic material with an ultrasmall spherical inclusion need to be reduced to those derived based on the classical poroelasticity theory. Setting the nonlocal parameter equal to zero in Equation (20) yields

(22) $$\left(\lambda +2\mu \right)\left(\frac{\partial^{2}u_{r}}{\partial r^{2}}+\frac{2}{r}\frac{\partial u_{r}}{\partial r}-\frac{2}{r^{2}}u_{r}\right)-\frac{\partial p}{\partial r}=0.$$

On the other hand, the first derivative of the volumetric strain with respect to the radial distance is obtained from Equation (19) as

(23) $$\frac{\partial \varepsilon }{\partial r}=\frac{\partial }{\partial r}\left(\varepsilon_{rr}+\varepsilon_{\theta \theta }+\varepsilon_{\phi \phi }\right)=\frac{\partial^{2}u_{r}}{\partial r^{2}}+\frac{2}{r}\frac{\partial u_{r}}{\partial r}-\frac{2}{r^{2}}u_{r}.$$

Substituting Equation (23) into Equation (22), one obtains

(24) $$\frac{\partial \varepsilon }{\partial r}=\frac{1}{\left(\lambda +2\mu \right)}\frac{\partial p}{\partial r}.$$

Equation (24), together with the mass balance Equation (21), are exactly the same as those widely reported in the literature for large-scale porous spherical inclusions using the classical poroelasticity [5].

## 3. Solution Procedure Using Galerkin Technique and PIM

To discretise the nonlocal scale-dependent governing equations using the Galerkin method, the radial displacement and fluid pressure are required to be approximated by a set of appropriate base functions that satisfy the boundary conditions. Consider a spherical inclusion of radius *R* embedded in a poroelastic medium under a uniform radial compression, as shown in Figure 1. From the symmetric condition, the radial displacement is zero at the inclusion centre, while it reaches its maximum value at the surface. By contrast, the fluid pressure is at its maximum at the centre, whereas it is equal to that of the background medium at the inclusion surface due to the continuity condition. Moreover, since the inclusion and its loading are symmetric around the centre, there is no fluid flow at the centre and, thus, the fluid pressure gradient is zero at that point. These boundary conditions can be written as

(25) $$\begin{array}{l} r=0:u_{r}=0,\\ r=R:u_{r}=u_{\max}, \end{array}$$

(26) $$\begin{array}{l} r=0:p=p_{\max},\\ r=R:p=p_{b},\\ r=0:\frac{\partial p}{\partial r}=0, \end{array}$$

in which $u_{max}$, $p_{max}$ and $p_{b}$ are the maximum radial displacement, maximum fluid pressure and the background pressure, respectively. In general, the nonlocal boundary conditions that are imposed on the displacement components of the inclusion, such as the radial displacement boundary conditions given by Equation (25), are the same as those of the classical poroelasticity model. However, nonlocal boundary conditions associated with stress components such as force resultants and moments deviate from their classical counterparts because of the effects of the nonlocal constitutive equations [13]. In this analysis, only stress nonlocality within the solid phase of the poroelastic medium is considered, and thus all boundary conditions related to the fluid phase, such as those specified by Equation (26), are the same as their corresponding classical boundary conditions.

Based on the boundary conditions given by Equations (25) and (26), the following expressions are suggested for the radial displacement and the fluid pressure inside the spherical inclusion

(27) $$u_{r}\left(r,t\right)=\sum_{m=1}^{M}u_{m}\left(t\right)\Psi_{m}\left(r\right)=\sum_{m=1}^{M}u_{m}\left(t\right)\sin\left[\frac{r}{R}\left(2m-1\right)\frac{\pi }{2}\right],$$

(28) $$p\left(r,t\right)=p_{b}\left(t\right)+\sum_{k=1}^{N}P_{k}\left(t\right)\Phi_{k}\left(r\right)=p_{b}\left(t\right)+\sum_{k=1}^{N}P_{k}\left(t\right)\cos\left[\frac{r}{R}\left(2k-1\right)\frac{\pi }{2}\right],$$

where $\Psi_{m}$ and $\Phi_{k}$ are the base functions of the radial displacement and fluid pressure, respectively. The number of base functions for the inclusion displacement and pressure are denoted by *M* and *N*, respectively. The proposed solution for the displacement and fluid pressure are not uniform, as can be seen from Equations (27) and (28). The second part of the solution on the right-hand side of these equations describes how radial displacement and fluid pressure change by the radial coordinate *r*. However, it is assumed that the loading condition on the surface of the inclusion is uniform. This assumption is made since the average size of the inclusion is very small compared to the background medium and, thus, the load is applied at a far distance from the inclusion. This makes the remote-load assumption valid and, hence, the loading condition on the inclusion surface is uniform according to the Eshelby theory [5,42]. In general, there are two types of widely used ultrasound elastography: (1) quasi-static and (2) dynamic. In the quasi-static technique, an external mechanical force is applied by a gradual compressive load, while in dynamic elastography, mechanical load is induced using vibrating probes or applying acoustic radiation forces [45]. In this analysis, the ultrasound elastography mode is of a quasi-static form, resulting in a static uniform load on the inclusion surface.

Substituting Equations (27) and (28) into Equations (20) and (21), multiplying both sides of each governing equation by its appropriate base function, and integrating over the whole volume of the spherical inclusion, the following discretised equations are obtained

(29) $$\begin{array}{l} \lambda \sum_{m=1}^{M}U_{m}\left[\int_{0}^{R}r^{2}\Psi_{m}\left(\frac{d^{2}\Psi_{m}}{dr^{2}}+\frac{2}{r}\frac{d\Psi_{m}}{dr}-\frac{2}{r^{2}}\Psi_{m}\right)dr\right]\\ +2\mu \sum_{m=1}^{M}U_{m}\left(\int_{0}^{R}r^{2}\Psi_{m}\frac{d^{2}\Psi_{m}}{dr^{2}}dr\right)+4\mu \sum_{m=1}^{M}U_{m}\left[\int_{0}^{R}r\Psi_{m}\left(\frac{d\Psi_{m}}{dr}-\frac{1}{r}\Psi_{m}\right)dr\right]\\ -\sum_{k=1}^{N}P_{k}\left(\int_{0}^{R}r^{2}\Psi_{m}\frac{d\Phi_{k}}{dr}dr\right)+{\left(e_{0}a_{c}\right)}^{2}\sum_{k=1}^{N}P_{k}\left[\int_{0}^{R}r^{2}\Psi_{m}\left(\frac{d^{3}\Phi_{k}}{dr^{3}}+\frac{2}{r}\frac{d^{2}\Phi_{k}}{dr^{2}}\right)dr\right]\\ -{\left(e_{0}a_{c}\right)}^{2}\left\{2\left(\lambda +2\mu \right)\sum_{m=1}^{M}U_{m}\left[\int_{0}^{R}r\Psi_{m}\left(\frac{d^{3}\Psi_{m}}{dr^{3}}+\frac{4}{r}\frac{d^{2}\Psi_{m}}{dr^{2}}\right)dr\right]\right.\\ +\left(\lambda +2\mu \right)\sum_{m=1}^{M}U_{m}\left[\int_{0}^{R}r^{2}\Psi_{m}\left(\frac{d^{4}\Psi_{m}}{dr^{4}}+\frac{4}{r}\frac{d^{3}\Psi_{m}}{dr^{3}}-\frac{4}{r^{2}}\frac{d^{2}\Psi_{m}}{dr^{2}}\right)dr\right]\\ -2\sum_{k=1}^{N}P_{k}\left(\int_{0}^{R}\Psi_{m}\frac{d\Phi_{k}}{dr}dr\right)+2{\left(e_{0}a_{c}\right)}^{2}\sum_{k=1}^{N}P_{k}\left[\int_{0}^{R}\Psi_{m}\left(\frac{d^{3}\Phi_{k}}{dr^{3}}+\frac{2}{r}\frac{d^{2}\Phi_{k}}{dr^{2}}\right)dr\right]\\ -4\sum_{k=1}^{N}P_{k}\left(\int_{0}^{R}r\Psi_{m}\frac{d^{2}\Phi_{k}}{dr^{2}}dr\right)+4{\left(e_{0}a_{c}\right)}^{2}\sum_{k=1}^{N}P_{k}\left[\int_{0}^{R}r\Psi_{m}\left(\frac{d^{4}\Phi_{k}}{dr^{4}}+\frac{2}{r}\frac{d^{3}\Phi_{k}}{dr^{3}}\right)dr\right]\\ -\sum_{k=1}^{N}P_{k}\left(\int_{0}^{R}r^{2}\Psi_{m}\frac{d^{3}\Phi_{k}}{dr^{3}}dr\right)-4{\left(e_{0}a_{c}\right)}^{2}\sum_{k=1}^{N}P_{k}\left(\int_{0}^{R}\Psi_{m}\frac{2}{r}\frac{d^{2}\Phi_{k}}{dr^{2}}dr\right)\\ +{\left(e_{0}a_{c}\right)}^{2}\sum_{k=1}^{N}P_{k}\left[\int_{0}^{R}r^{2}\Psi_{m}\left(\frac{d^{5}\Phi_{k}}{dr^{5}}+\frac{2}{r}\frac{d^{4}\Phi_{k}}{dr^{4}}\right)dr\right]\\ \left.-4{\left(e_{0}a_{c}\right)}^{2}\sum_{k=1}^{N}P_{k}\left[\int_{0}^{R}\Psi_{m}\left(\frac{d^{3}\Phi_{k}}{dr^{3}}-\frac{1}{r}\frac{d^{2}\Phi_{k}}{dr^{2}}\right)dr\right]\right\}\\ -2{\left(e_{0}a_{c}\right)}^{2}\left\{\left(\lambda +2\mu \right)\sum_{m=1}^{M}U_{m}\left[\int_{0}^{R}r\Psi_{m}\left(\frac{d^{3}\Psi_{m}}{dr^{3}}+\frac{4}{r}\frac{d^{2}\Psi_{m}}{dr^{2}}\right)dr\right]\right.\\ -2\sum_{k=1}^{N}P_{k}\left(\int_{0}^{R}\Psi_{m}\frac{d\Phi_{k}}{dr}dr\right)-\sum_{k=1}^{N}P_{k}\left(\int_{0}^{R}r\Psi_{m}\frac{d^{2}\Phi_{k}}{dr^{2}}dr\right)\\ +2{\left(e_{0}a_{c}\right)}^{2}\sum_{k=1}^{N}P_{k}\left[\int_{0}^{R}\Psi_{m}\left(\frac{d^{3}\Phi_{k}}{dr^{3}}+\frac{2}{r}\frac{d^{2}\Phi_{k}}{dr^{2}}\right)dr\right]\\ \left.+{\left(e_{0}a_{c}\right)}^{2}\sum_{k=1}^{N}P_{k}\left[\int_{0}^{R}r\Psi_{m}\left(\frac{d^{4}\Phi_{k}}{dr^{4}}+\frac{2}{r}\frac{d^{3}\Phi_{k}}{dr^{3}}-\frac{2}{r^{2}}\frac{d^{2}\Phi_{k}}{dr^{2}}\right)dr\right]\right\}\\ +8{\left(e_{0}a_{c}\right)}^{2}\mu \sum_{m=1}^{M}U_{m}\left(\int_{0}^{R}\Psi_{m}\frac{d^{2}\Psi_{m}}{dr^{2}}dr\right)-2{\left(e_{0}a_{c}\right)}^{2}\sum_{k=1}^{N}P_{k}\left(\int_{0}^{R}\Psi_{m}\frac{d\Phi_{k}}{dr}dr\right)\\ +2{\left(e_{0}a_{c}\right)}^{4}\sum_{k=1}^{N}P_{k}\left[\int_{0}^{R}\Psi_{m}\left(\frac{d^{3}\Phi_{k}}{dr^{3}}+\frac{2}{r}\frac{d^{2}\Phi_{k}}{dr^{2}}\right)dr\right]=0, \end{array}$$

(30) $$\begin{array}{l} \sum_{m=1}^{M}\frac{dU_{m}}{dt}\left[\int_{0}^{R}r^{2}\Phi_{k}\left(\frac{d\Psi_{m}}{dr}+\frac{2}{r}\Psi_{m}\right)dr\right]\\ +\chi_{tot}p_{b}\int_{0}^{R}r^{2}\Phi_{k}dr+\chi_{tot}\sum_{k=1}^{N}P_{k}\left(\int_{0}^{R}r^{2}{\left(\Phi_{k}\right)}^{2}dr\right)\\ =\frac{\lambda_{hc}}{\gamma_{vw}}\sum_{k=1}^{N}P_{k}\left[\int_{0}^{R}r^{2}\Phi_{k}\left(\frac{d^{2}\Phi_{k}}{dr^{2}}+\frac{2}{r}\frac{d\Phi_{k}}{dr}\right)dr\right]. \end{array}$$

In general, the volume element in the spherical coordinate system is $dV=r^{2}sin\varphi drd\theta d\varphi$, where φ and θ are the azimuthal and polar angles, respectively. Due to the symmetry of the problem, the element volume used to perform the integration over the inclusion body in the above discretised equations is ${dV}_{sym}=r^{2}dr$. The fluid pressure of the background at the interface is assumed to be zero ($p_{b}=0$) [5]. To avoid any numerical error caused by unscaled features and parameters, a set of dimensionless parameters is introduced by

(31) $$\begin{array}{l} \zeta =\frac{r}{R},{\bar{\gamma }}_{nl}=\frac{\gamma_{nl}}{R^{2}},\gamma_{nl}=\frac{e_{0}a_{c}}{R},\bar{G}=\frac{G}{H_{aG}},{\bar{U}}_{j}=\frac{U_{j}}{R},\\ {\bar{P}}_{j}=\frac{P_{j}}{H_{ag}},\bar{t}=\frac{\lambda_{hc}G}{\gamma_{vw}R^{2}}t,{\bar{\chi }}_{tot}=\frac{\gamma_{vw}R^{2}}{\lambda_{hc}}\chi_{tot},H_{aG}=K+\frac{4}{3}G. \end{array}$$

Here, *H_ag_* is the aggregate modulus of the spherical inclusion. The discretised Equations (29) and (30) can be written in a compact way, as follows

(32) $$\sum_{m=1}^{M}C_{i,m}^{\left(1Ub\right)}{\bar{U}}_{m}+\sum_{k=1}^{N}C_{i,k}^{\left(1Pb\right)}{\bar{P}}_{k}=0,$$

(33) $$\sum_{m=1}^{M}C_{j,m}^{\left(2Ud\right)}\frac{d{\bar{U}}_{m}}{d\bar{t}}+\sum_{k=1}^{N}C_{j,k}^{\left(2Pb\right)}{\bar{P}}_{k}=0,$$

where $C_{i,m}^{\left(1Ub\right)}$, $C_{i,k}^{\left(1Pb\right)}$, $C_{j,m}^{\left(2Ud\right)}$ and $C_{j,k}^{\left(2Pb\right)}$ are calculated using Equations (29) and (30) by performing all integrations over the inclusion body. For the sake of convenience, Equations (32) and (33) can be expressed in a matrix form as

(34) $$Ay_{1}+By_{2}=0,$$

(35) $$C\frac{dy_{1}}{d\bar{t}}+Fy_{2}=0,$$

where

(36) $$\begin{array}{l} y_{1}=\left\{{\bar{U}}_{m}\right\},y_{2}=\left\{{\bar{P}}_{k}\right\},A=\left[C_{i,m}^{\left(1Ub\right)}\right],\\ B=\left[C_{i,k}^{\left(1Pb\right)}\right],C=\left[C_{j,m}^{\left(2Ud\right)}\right],F=\left[C_{j,k}^{\left(2Pb\right)}\right]. \end{array}$$

Equations (34) and (35) give a set of time-dependent ordinary differential equations in a matrix form. From these two equations, one can obtain

(37) $$y_{1}=-A^{-1}B\left[\exp\left(-H^{-1}F\bar{t}\right)\right]y_{20},$$

(38) $$y_{2}=\left[\exp\left(-H^{-1}F\bar{t}\right)\right]y_{20},$$

in which $y_{20}$ is the initial value of the vector $y_{2}$ at $\bar{t}=0$. Matrix **H** is defined by

(39) $$H=-CA^{-1}B.$$

Based on the procedure used in the precise integration method (PIM), a time step dimensionless parameter η is introduced by [46,47]

(40) $${\bar{t}}_{0}=0,{\bar{t}}_{1}=\eta ,{\bar{t}}_{2}=2\eta ,{\bar{t}}_{3}=3\eta ,\cdots {\bar{t}}_{k}=k\eta .$$

Using Equation (40), the vector of dimensionless pressure ${\bar{P}}_{k}$ is calculated as

(41) $$\begin{array}{l} {\left(y_{2}\right)}_{1}=y_{2}\left(\bar{t}={\bar{t}}_{1}\right)=\left[\exp\left(-W\eta \right)\right]y_{20}=Ty_{20},\\ {\left(y_{2}\right)}_{2}=y_{2}\left(\bar{t}={\bar{t}}_{2}\right)=\left[\exp\left(-W\eta \right)\right]\exp\left(-W\eta \right)y_{20}=T{\left(y_{2}\right)}_{1},\\ \vdots \\ {\left(y_{2}\right)}_{k}=y_{2}\left(\bar{t}={\bar{t}}_{k}\right)=\left[\exp\left(-W\eta \right)\right]\exp\left(-W(k-1)\eta \right)y_{20}=T{\left(y_{2}\right)}_{k-1}, \end{array}$$

where

(42) $$W=H^{-1}F,T=\exp\left(-W\eta \right)={\left[\exp\left(-W\tau_{p}\right)\right]}^{m_{p}},$$

and

(43) $$\tau_{p}=\frac{\eta }{m_{p}},m_{p}=2^{N_{p}},$$

The recommended value for the $N_{p}$ is twenty [47], which is commonly utilised in PIM. From Equation (43) and by adopting such a big value for $m_{p}$, it is found that the new time interval $\tau_{p}$ is very small. Thus, the following approximation of the exponential function is valid by employing the Taylor expansion

(44) $$\exp\left(-W\tau_{p}\right)\approx I+\Lambda_{p},$$

where

(45) $$\Lambda_{p}=-W\tau_{p}+\frac{1}{2}{\left(W\tau_{p}\right)}^{2}\left[I-\frac{1}{3}\left(W\tau_{p}\right)+\frac{1}{12}{\left(W\tau_{p}\right)}^{2}\right].$$

In Equation (44), I denotes the identity matrix. In view of Equations (44) and (42), we have

(46) $$\begin{array}{l} N_{p}=1:T={\left(I+\Lambda_{p}\right)}^{2}=I+T_{1},\;T_{1}=2\Lambda_{p}+{\left(\Lambda_{p}\right)}^{2},\\ N_{p}=2:T={\left(I+\Lambda_{p}\right)}^{4}={\left(I+T_{1}\right)}^{2}=I+T_{2},\;T_{2}=2T_{1}+{\left(T_{1}\right)}^{2},\\ N_{p}=3:T={\left(I+\Lambda_{p}\right)}^{8}={\left(I+T_{2}\right)}^{2}=I+T_{3},\;T_{3}=2T_{2}+{\left(T_{2}\right)}^{2},\\ \vdots \\ N_{p}=k:T={\left(I+\Lambda_{p}\right)}^{2^{k}}={\left(I+T_{k-1}\right)}^{2}=I+T_{k},\;T_{k}=2T_{k-1}+{\left(T_{k-1}\right)}^{2}. \end{array}$$

The time-dependent part of the fluid pressure of the spherical inclusion is obtained by substituting Equation (46) into Equation (41). The time-dependent part of the radial displacement can be calculated by Equation (37). The resultant time-dependent parts are then substituted into Equations (27) and (28) to obtain the final solution.

## 4. Analytical Solution for One Galerkin Term

An analytical solution can be calculated for the simplest case where one Galerkin term is assumed for both the radial displacement and the fluid pressure of the inclusion. Using Equations (27) and (28), we have

(47) $${\bar{u}}_{r}\left(\zeta ,\bar{t}\right)=\bar{U}\left(\bar{t}\right)\Psi \left(\zeta \right)=\bar{U}\left(\bar{t}\right)\sin\left(\frac{\pi }{2}\zeta \right),$$

(48) $$\bar{p}\left(\zeta ,\bar{t}\right)=\bar{P}\left(\bar{t}\right)\Phi \left(\zeta \right)=\bar{P}\left(\bar{t}\right)\cos\left(\frac{\pi }{2}\zeta \right).$$

Substituting Equations (47) and (48) into the mass balance and equilibrium equations, and using the Galerkin technique, the resultant time-dependent equations are

(49) $${\tilde{C}}_{1}\bar{U}\left(\bar{t}\right)+{\tilde{C}}_{2}\bar{P}\left(\bar{t}\right)=0,$$

(50) $${\tilde{C}}_{3}\frac{d\bar{U}(\bar{t})}{d\bar{t}}+{\tilde{C}}_{4}\bar{P}(\bar{t})=0,$$

where

$$\begin{array}{l} {\tilde{C}}_{1}=\left[2+\pi^{2}{\bar{\gamma }}_{nl}\left(2\bar{G}-3\right)\right]\int_{0}^{1}\Psi^{2}d\zeta -\pi \left(1+\pi^{2}{\bar{\gamma }}_{nl}\right)\int_{0}^{1}\zeta \Psi \Phi d\zeta \\ +{\left(\frac{\pi }{2}\right)}^{2}\left[1+{\bar{\gamma }}_{nl}{\left(\frac{\pi }{2}\right)}^{2}\right]\int_{0}^{1}\zeta^{2}\Psi^{2}d\zeta ,\\ {\tilde{C}}_{2}=-\left(\frac{\pi }{2}\right)\left[1+2{\left(\frac{\pi }{2}\right)}^{2}{\bar{\gamma }}_{nl}+{\left(\frac{\pi }{2}\right)}^{4}{\bar{\gamma }}_{nl}^{2}\right]\int_{0}^{1}\zeta^{2}\Psi^{2}d\zeta \\ +2\pi {\bar{\gamma }}_{nl}\left[1+3{\left(\frac{\pi }{2}\right)}^{2}{\bar{\gamma }}_{nl}\right]\int_{0}^{1}\Psi^{2}d\zeta \\ +8{\bar{\gamma }}_{nl}{\left(\frac{\pi }{2}\right)}^{2}\left(1+{\left(\frac{\pi }{2}\right)}^{2}{\bar{\gamma }}_{nl}\right)\int_{0}^{1}\zeta \Psi \Phi d\zeta , \end{array}$$

(51) $$\begin{array}{l} {\tilde{C}}_{3}=\left(\frac{\pi }{2}\right)\int_{0}^{1}\zeta^{2}\cos^{2}\left(\frac{\pi }{2}\zeta \right)d\zeta +\int_{0}^{1}\zeta \sin\left(\pi \zeta \right)d\zeta ,\\ {\tilde{C}}_{4}=\frac{1}{\bar{G}}\left[\left({\bar{\chi }}_{tot}+{\left(\frac{\pi }{2}\right)}^{2}\right)\int_{0}^{1}\zeta^{2}\cos^{2}\left(\frac{\pi }{2}\zeta \right)d\zeta +\left(\frac{\pi }{2}\right)\int_{0}^{1}\zeta \sin\left(\pi \zeta \right)d\zeta \right]. \end{array}$$

Substituting Equation (49) into Equation (50), and then solving the resultant time-dependent ordinary equation, one obtains

(52) $$\begin{array}{l} \bar{U}\left(\bar{t}\right)={\bar{U}}_{0}\exp\left(-{\tilde{\Theta }}_{tr}\bar{t}\right),\\ \bar{P}\left(\bar{t}\right)={\bar{P}}_{0}\exp\left(-{\tilde{\Theta }}_{tr}\bar{t}\right), \end{array}$$

In which ${\tilde{\Theta }}_{tr}=-{\tilde{C}}_{1}{\tilde{C}}_{4}/{\tilde{C}}_{2}{\tilde{C}}_{3}$. Using Equations (47), (48) and (52), together with the definition of dimensionless parameters given by Equation (31), the radial displacement and fluid pressure of the spherical inclusion are

(53) $$U_{r}\left(r,t\right)=U_{0}\sin\left(\frac{\pi }{2}\frac{r}{R}\right)\exp\left(-{\tilde{\Theta }}_{tr}\frac{\lambda_{hc}G}{\Upsilon_{vw}R^{2}}t\right),$$

(54) $$p\left(r,t\right)=H_{aG}\bar{P}\Phi =P_{0}\cos\left(\frac{\pi }{2}\frac{r}{R}\right)\exp\left(-{\tilde{\Theta }}_{tr}\frac{\lambda_{hc}G}{\Upsilon_{vw}R^{2}}t\right).$$

## 5. Dormand–Prince Technique for Two Galerkin Terms

In this section, an approximate solution is given for the radial displacement and fluid pressure inside the ultrasmall inclusion by assuming two Galerkin terms and using the Dormand–Prince technique [48]. This method is a numerical embedded technique from the Runge–Kutta family for solving differential equations of ordinary types. To accurately extract the fourth- and fifth-order solutions, the Dormand–Prince method utilises a six-function evaluation approach. Using Equations (27) and (28), the two Galerkin-term approximation leads to

(55) $$\begin{array}{l} {\bar{u}}_{r}\left(\zeta ,\bar{t}\right)={\bar{U}}_{1}\left(\bar{t}\right)\Psi_{1}\left(\zeta \right)+{\bar{U}}_{2}\left(\bar{t}\right)\Psi_{2}\left(\zeta \right)\\ ={\bar{U}}_{1}\left(\bar{t}\right)\sin\left(\frac{\pi }{2}\zeta \right)+{\bar{U}}_{2}\left(\bar{t}\right)\sin\left(\frac{3\pi }{2}\zeta \right), \end{array}$$

(56) $$\begin{array}{l} \bar{p}\left(\zeta ,\bar{t}\right)={\bar{P}}_{1}\left(\bar{t}\right)\Phi_{1}\left(\zeta \right)+{\bar{P}}_{2}\left(\bar{t}\right)\Phi_{2}\left(\zeta \right)\\ ={\bar{P}}_{1}\left(\bar{t}\right)\cos\left(\frac{\pi }{2}\zeta \right)+{\bar{P}}_{2}\left(\bar{t}\right)\cos\left(\frac{3\pi }{2}\zeta \right). \end{array}$$

Substituting Equations (55) and (56) into the governing equations of the spherical inclusion, multiplying the resultant equations by their corresponding base functions and integrating over the whole inclusion body, the time-dependent discretised equations are

(57) $$\left[\begin{array}{cc} C_{1,1}^{\left(1Ub\right)} & C_{1,2}^{\left(1Ub\right)}\\ C_{2,1}^{\left(1Ub\right)} & C_{2,2}^{\left(1Ub\right)} \end{array}\right]\left\{\begin{array}{c} {\bar{U}}_{1}\left(\bar{t}\right)\\ {\bar{U}}_{2}\left(\bar{t}\right) \end{array}\right\}+\left[\begin{array}{cc} C_{1,1}^{\left(1Pb\right)} & C_{1,2}^{\left(1Pb\right)}\\ C_{2,1}^{\left(1Pb\right)} & C_{2,2}^{\left(1Pb\right)} \end{array}\right]\left\{\begin{array}{c} {\bar{P}}_{1}\left(\bar{t}\right)\\ {\bar{P}}_{2}\left(\bar{t}\right) \end{array}\right\}=0,$$

(58) $$\left[\begin{array}{cc} C_{1,1}^{\left(2Ud\right)} & C_{1,2}^{\left(2Ud\right)}\\ C_{2,1}^{\left(2Ud\right)} & C_{2,2}^{\left(2Ud\right)} \end{array}\right]\left\{\begin{array}{c} \frac{d{\bar{U}}_{1}\left(\bar{t}\right)}{d\bar{t}}\\ \frac{d{\bar{U}}_{2}\left(\bar{t}\right)}{d\bar{t}} \end{array}\right\}+\left[\begin{array}{cc} C_{1,1}^{\left(2Pb\right)} & C_{1,2}^{\left(2Pb\right)}\\ C_{2,1}^{\left(2Pb\right)} & C_{2,2}^{\left(2Pb\right)} \end{array}\right]\left\{\begin{array}{c} {\bar{P}}_{1}\left(\bar{t}\right)\\ {\bar{P}}_{2}\left(\bar{t}\right) \end{array}\right\}=0.$$

Obtaining the dimensionless vector of time-dependent radial displacements and substituting them into Equation (58) leads to

(59) $$\left\{\begin{array}{c} \frac{d{\bar{P}}_{1}\left(\bar{t}\right)}{d\bar{t}}\\ \frac{d{\bar{P}}_{2}\left(\bar{t}\right)}{d\bar{t}} \end{array}\right\}=\left[\begin{array}{cc} C_{1,1}^{\left(4Pd\right)} & C_{1,2}^{\left(4Pd\right)}\\ C_{2,1}^{\left(4Pd\right)} & C_{2,2}^{\left(4Pd\right)} \end{array}\right]\left\{\begin{array}{c} {\bar{P}}_{1}\\ {\bar{P}}_{2} \end{array}\right\},$$

where

(60) $$\left[\begin{array}{cc} C_{1,1}^{\left(4Pd\right)} & C_{1,2}^{\left(4Pd\right)}\\ C_{2,1}^{\left(4Pd\right)} & C_{2,2}^{\left(4Pd\right)} \end{array}\right]=-{\left[\begin{array}{cc} C_{1,1}^{\left(3Pd\right)} & C_{1,2}^{\left(3Pd\right)}\\ C_{2,1}^{\left(3Pd\right)} & C_{2,2}^{\left(3Pd\right)} \end{array}\right]}^{-1}\left[\begin{array}{cc} C_{1,1}^{\left(2Pb\right)} & C_{1,2}^{\left(2Pb\right)}\\ C_{2,1}^{\left(2Pb\right)} & C_{2,2}^{\left(2Pb\right)} \end{array}\right],$$

(61) $$\left[\begin{array}{cc} C_{1,1}^{\left(3Pd\right)} & C_{1,2}^{\left(3Pd\right)}\\ C_{2,1}^{\left(3Pd\right)} & C_{2,2}^{\left(3Pd\right)} \end{array}\right]=-\left[\begin{array}{cc} C_{1,1}^{\left(2Ud\right)} & C_{1,2}^{\left(2Ud\right)}\\ C_{2,1}^{\left(2Ud\right)} & C_{2,2}^{\left(2Ud\right)} \end{array}\right]{\left[\begin{array}{cc} C_{1,1}^{\left(1Ub\right)} & C_{1,2}^{\left(1Ub\right)}\\ C_{2,1}^{\left(1Ub\right)} & C_{2,2}^{\left(1Ub\right)} \end{array}\right]}^{-1}\left[\begin{array}{cc} C_{1,1}^{\left(1Pb\right)} & C_{1,2}^{\left(1Pb\right)}\\ C_{2,1}^{\left(1Pb\right)} & C_{2,2}^{\left(1Pb\right)} \end{array}\right].$$

The vector of dimensionless fluid pressure of the spherical inclusion is calculated by writing a Matlab code using the Dormand–Prince technique. Similar numerical solutions can also be developed for more than two Galerkin terms.

## 6. Analytical Solution for Local Large-Scale Spherical Inclusions

For the sake of comparison and validation, an analytical solution is obtained for local large-scale spherical inclusions where there are no scale effects. Setting the nonlocal parameter equal to zero ($e_{0}a_{c}=0$), the governing equations of the inclusion are reduced to

(62) $$\frac{\partial \varepsilon }{\partial r}=\frac{1}{H_{ag}}\frac{\partial p}{\partial r},$$

(63) $$\frac{\partial \varepsilon }{\partial t}+\chi_{tot}p=\frac{\lambda_{hc}}{\gamma_{vw}}\nabla^{2}p.$$

Integrating both sides of Equation (62) with respect to the radial coordinate parameter *r*, the volumetric strain is obtained by

(64) $$\varepsilon =\frac{1}{H_{ag}}\left(p+c_{eq}\right),$$

where $c_{eq}$ denotes the integration constant that is generally related to the initial condition. For simplification and ease of use, the subscripts “*tot*” and “*ag*” are dropped from the microfiltration coefficient and aggregate modulus (i.e., $\chi_{tot}=\chi$ and $H_{ag}=H$), respectively. The volumetric strain, fluid pressure and the integration constant are [5]

(65) $$\begin{array}{l} \varepsilon \left(r,t\right)=\varepsilon^{\prime }\left(r,t\right)e^{-H\chi t},\\ p\left(r,t\right)=p^{\prime }\left(r,t\right)e^{-H\chi t},\\ c_{eq}\left(t\right)={c^{\prime }}_{eq}\left(t\right)e^{-H\chi t}. \end{array}$$

Substituting Equation (65) into Equation (64) leads to

(66) $$\varepsilon^{\prime }=\frac{1}{H}\left(p^{\prime }+{c^{\prime }}_{eq}\right).$$

The second and first derivatives of Equation (66) with respect to *r* are

(67) $$\begin{array}{l} H\frac{\partial \varepsilon^{\prime }}{\partial r}=\frac{\partial p^{\prime }}{\partial r}\\ H\frac{\partial^{2}\varepsilon^{\prime }}{\partial r^{2}}=\frac{\partial^{2}p^{\prime }}{\partial r^{2}}. \end{array}$$

Using Equations (63), (66) and (67), the following relation is obtained for the spherical inclusion

(68) $$\frac{\partial \varepsilon^{\prime }}{\partial t}-\chi {c^{\prime }}_{eq}=\frac{\lambda_{hc}H}{\gamma_{vw}}\left(\frac{\partial^{2}\varepsilon^{\prime }}{\partial r^{2}}+\frac{2}{r}\frac{\partial \varepsilon^{\prime }}{\partial r}\right).$$

The integration constant is affected by the initial conditions, and they can be taken in a way that this constant becomes zero (${c^{\prime }}_{eq}=0$) [5]. Let us define two dimensionless parameters as ζ=r/R and $\tau^{\prime }=\lambda_{hc}Ht/\left(R^{2}\gamma_{vw}\right)$. Using these definitions, Equation (68) is expressed by

(69) $$\frac{\partial \varepsilon^{\prime }}{\partial \tau^{\prime }}=\frac{\partial^{2}\varepsilon^{\prime }}{\partial \zeta^{2}}+\frac{2}{\zeta }\frac{\partial \varepsilon^{\prime }}{\partial \zeta }.$$

Now, a new parameter is introduced for the sake of convenience as

(70) $$\varepsilon^{\prime }\left(\zeta ,\tau^{\prime }\right)=\frac{1}{\zeta }\Sigma \left(\zeta ,\tau^{\prime }\right).$$

Equation (70) is used to change the variable in Equation (69) as

(71) $$\frac{\partial \Sigma }{\partial \tau^{\prime }}=\frac{\partial^{2}\Sigma }{\partial \zeta^{2}}.$$

Performing the Laplace transform on both sides of Equation (71) leads to the following equation in the s−ζ domain

(72) $$s\bar{\Sigma }=\frac{\partial^{2}\bar{\Sigma }}{\partial \zeta^{2}},$$

where

(73) $$\bar{\Sigma }(s)=L\left(\Sigma (\tau^{\prime })\right)=\int_{0}^{\infty }\Sigma (\tau^{\prime })e^{-s\tau^{\prime }}d\tau^{\prime }.$$

It is observed that the Laplace transform in conjunction with the change in variables results in a less complex differential equation that is easier to be solved analytically. The solution of Equation (72) can be written as

(74) $$\bar{\Sigma }\left(s\right)=C_{1}\left(s\right)\cosh\left(\sqrt{s}\zeta \right)+C_{2}\left(s\right)\sinh\left(\sqrt{s}\zeta \right),$$

where *C*_1_ and *C*_2_ depend only on *s* and are obtained by using the boundary conditions. Substituting Equation (74) into Equation (70), we have

(75) $$\bar{\varepsilon^{\prime }}\left(\zeta ,s\right)=\frac{1}{\zeta }\bar{\Sigma }\left(\zeta ,s\right)=\frac{1}{\zeta }\left[C_{1}\left(s\right)\cosh\left(\sqrt{s}\zeta \right)+C_{2}\left(s\right)\sinh\left(\sqrt{s}\zeta \right)\right].$$

Using the first relation of Equation (65), together with the definition of dimensionless time ($\tau^{\prime }$), the following relation is obtained between $\varepsilon^{\prime }$ and ε

(76) $$\varepsilon \left(\zeta ,\tau^{\prime }\right)=\varepsilon^{\prime }\left(\zeta ,\tau^{\prime }\right)e^{-Q\tau^{\prime }},Q=\frac{R^{2}\gamma_{vw}\chi }{\lambda_{hc}}.$$

Taking the Laplace transform of Equation (76) leads to

(77) $$\bar{\varepsilon }=L(\varepsilon )=\int_{0}^{\infty }\varepsilon^{\prime }\left(\zeta ,\tau^{\prime }\right)e^{-\left(Q+s\right)\tau^{\prime }}d\tau^{\prime }={\bar{\varepsilon }}^{\prime }\left(\zeta ,s+Q\right).$$

To calculate the coefficients of $\bar{\Sigma }\left(s\right)$, namely *C*_1_ and *C*_2_, the boundary conditions of the spherical inclusion are used as follows [49]

(78) $$\begin{array}{l} \zeta =1:\varepsilon \left(1,t\right)=\varepsilon^{\prime }\left(1,t\right)e^{-H\chi t},\\ \varepsilon \left(1,t\right)-\frac{2\left(1-2v\right)}{\left(1-v\right)}\int_{0}^{1}\zeta^{2}\varepsilon \left(\zeta ,t\right)d\zeta =-\frac{\left(1+v\right)\left(1-2v\right)}{\left(1-v\right)}\frac{\sigma_{bc}}{E}, \end{array}$$

and

(79) $$\zeta =0:\varepsilon \left(0,\tau^{\prime }\right)=\varepsilon^{\prime }\left(0,t\right)e^{-H\chi t}<<\infty .$$

Here v, E and $\sigma_{bc}$ are Poisson ratio, Young’s modulus and stress on the spherical inclusion surface, respectively. Taking the Laplace transform of Equations (78) and (79), and substituting Equations (75)–(77) into the resultant relations of the boundary conditions, one can obtain

(80) $$\begin{array}{l} C_{2}\left(s\right)=-\frac{1}{\left\{\left[\left(1-v\right)s+2\left(1-2v\right)\right]\sinh\left(\sqrt{s}\right)-2\left(1-2v\right)\sqrt{s}\cosh\left(\sqrt{s}\right)\right\}}\\ \times \frac{s\left(1+v\right)\left(1-2v\right)}{\left(s-Q\right)}\frac{\sigma_{bc}}{E},C_{1}\left(s\right)=0. \end{array}$$

Substituting Equation (80) into Equation (75) leads to the following relation

(81) $$\begin{array}{l} \bar{\varepsilon^{\prime }}\left(\zeta ,s\right)=-\frac{1}{\left\{\left[\left(1-v\right)s+2\left(1-2v\right)\right]\sinh\left(\sqrt{s}\right)-2\left(1-2v\right)\sqrt{s}\cosh\left(\sqrt{s}\right)\right\}}\\ \times \frac{\sigma_{bc}}{E}\frac{\left(1+v\right)\left(1-2v\right)s}{\left(s-Q\right)}\frac{1}{\zeta }\sinh\left(\sqrt{s}\zeta \right). \end{array}$$

Using the complex inversion integral [40], the Laplace transform inverse of Equation (81) can be calculated as

(82) $$\begin{array}{l} Q=0:\varepsilon \left(r,t\right)=-k_{s0}\left(1-2v\right)\frac{\sigma_{bc}}{E}\left\{1+\frac{4\left(1+v\right)\left(1-2v\right)}{k_{s0}\left(\frac{r}{R}\right)}\times \right.\\ \left.\sum_{n=1}^{\infty }\frac{1}{\left[2\left(1+v\right)\left(1-2v\right)-{\left(1-v\right)}^{2}x_{n}\right]}\frac{\sin\left(\sqrt{x_{n}}\frac{r}{R}\right)}{\sin\left(\sqrt{x_{n}}\right)}\exp\left(-x_{n}\frac{\lambda_{hc}H}{R^{2}\gamma_{vw}}t\right)\right\}, \end{array}$$

(83) $$\begin{array}{l} Q\neq 0:\varepsilon \left(r,t\right)=-\frac{\sigma_{bc}\left(1+v\right)\left(1-2v\right)Q\sinh\left(\sqrt{Q}\frac{r}{R}\right)}{\frac{r}{R}E\left\{\left[\left(1-v\right)Q+2\left(1-2v\right)\right]\sinh\left(\sqrt{Q}\right)-2\left(1-2v\right)\sqrt{Q}\cosh\left(\sqrt{Q}\right)\right\}}\\ -\sum_{n=1}^{\infty }\frac{4\sigma_{bc}{\left(1-2v\right)}^{2}\left(1+v\right)x_{n}}{E\left[2\left(1+v\right)\left(1-2v\right)-{\left(1-v\right)}^{2}x_{n}\right]\left(x_{n}+Q\right)}\frac{\sin\left(\sqrt{x_{n}}\frac{r}{R}\right)}{\frac{r}{R}\sin\left(\sqrt{x_{n}}\right)}\exp\left[-\left(x_{n}+Q\right)\frac{\lambda_{hc}H}{R^{2}\gamma_{vw}}t\right], \end{array}$$

where $x_{n}$ is obtained from the following relation

(84) $$\tan\left(\sqrt{x_{n}}\right)=\frac{2\left(1-2v\right)\sqrt{x_{n}}}{\left[2\left(1-2v\right)-\left(1-v\right)x_{n}\right]}.$$

## 7. Integration of Nonlocal Poroelastic Model with Light Gradient Boosting Machine

The nonlocal poroelastic model has been developed based on some assumptions and limitations including material linearity, spherical shapes for inclusions and small ratios of inclusion radius to poroelastic medium length. However, in practical applications, a violation of at least one of these assumptions could happen, which restricts the application of the scale-dependent nonlocal poroelastic model. Overcoming all the limitations of the above nonlocal model by the use of nonlinear nonlocal poroelasticity is either impossible or comes with significant mathematical challenges and computational costs. Integration of the nonlocal continuum model of poroelasticity with a light gradient boosting machine (LGBM) enables greater flexibility for extracting patterns in experimental and computational data, as well as for incorporating additional effects such as nonlinearity and geometrical imperfections. The LGBM is an open source, fast and efficient gradient boosting framework developed by Microsoft [50] that has been recently used for many machine learning tasks in various applications [51,52,53]. Its high speed, lower memory usage and efficient performance, particularly when working on large-scale datasets, make this machine learning algorithm an ideal candidate to be integrated with the nonlocal poroelastic model. Another reason for suitability of the LGBM is the capability of handling both regression and classification problems. Inclusion models are often used to detect imperfections and abnormalities such as solid tumours, in which both classifications and regression tasks might be needed.

In the LGBM, a strong predictive model is created by the combination of several weak estimators (decision trees). The estimators are developed sequentially, in which each estimator tries to correct the errors caused by the previous ensembled decision trees. A leaf-wise tree growth approach is used, in which only leaves with maximum reduction in the loss function are chosen to expand the decision tree. Compared to level-wise tree growth, this approach generally leads to lower loss values and higher accuracies. However, leaf-wise tree growth algorithms are more prone to overfitting, especially on small datasets.

In this analysis, three different types of boosting strategies are utilised for the LGBM integrated with the nonlocal continuum model: (1) gradient-based one-side sampling (GOSS), (2) dropouts meet multiple additive regression trees (DART) and (3) traditional gradient boosting decision tree (GBDT). The GOSS utilises a subsampling procedure to place more emphasis on subsamples with higher gradients. In fact, subsamples with high gradients play a more significant role in building decision trees. In addition to the general advantages of subsampling such as variety introduction, rapid training process and less chance of overfitting, GOSS-based subsampling benefits from improved efficiency, less memory usage and faster convergence. On the other hand, the DART boosting algorithm addresses the problem of over-specialisation by employing the idea of dropouts from deep learning. During each iteration, random dropouts are conducted to avoid over-reliance on earlier trees and improve the generalisation of the model.

**Figure 2:**
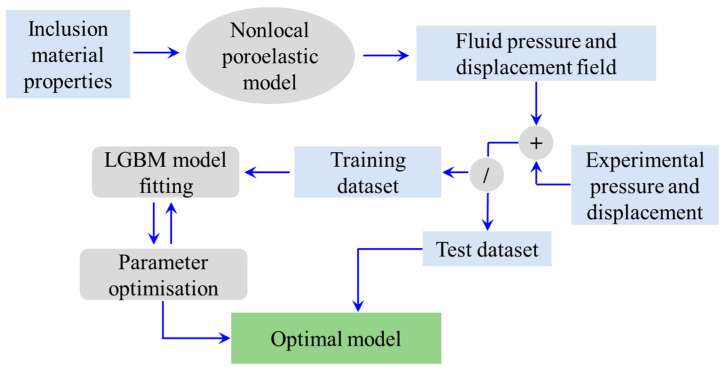
Integration of the nonlocal poroelasticity model with the light gradient boosting machine learning to predict the mechanical characteristics of small-scale spherical inclusions. First, the material properties of the inclusion are given to the nonlocal poroelastic model to obtain the fluid pressure and displacement field (theoretical data). If experimental observations are available, they are recommended to be added to the training and test datasets to account for additional complexities in the mechanics of poroelastic inclusions. At the next step, the collected dataset is divided into two subsets for training and testing. A common approach is to use 70% of data for training (i.e., LGBM model fitting) and the rest for an accuracy test. During the model fitting, parameter optimisation is conducted to obtain the optimal LGBM model with minimum error.

Figure 2 shows the required steps involved in the integration of the nonlocal poro-elasticity model and the LGBM algorithm of machine learning. First, inclusion features such as average radius, nonlocal scale coefficient, elastic modulus, Poisson’s ratio and hydraulic conductivity, as well as the times of interest, are given to the nonlocal scale-dependent model of poroelasticity, and the inclusion’s pressure and radial displacement are obtained. The calculated fluid pressures and displacements are then employed to build a training dataset for fitting the LGBM model. Depending on the availability of experimental tools and measurements, empirical observations can also be supplied, leading to a more robust and accurate hybrid model of poroelastic inclusions that would be capable of incorporating additional effects such as nonlinearity and geometrical imperfections. Overall, nonlocal poroelasticity results account for the underlying physics of the inclusion problem, while the experimental data could help incorporate the violation of any assumption made in the nonlocal continuum modelling.

In this study, the light gradient boosting machine learning model is developed using open-source python libraries including scikit-learn 1.2.2, pandas version 1.5.3, lightGBM 3.3.5 and NumPy 1.24.3. The scaling process is performed on numerical features such as inclusion radius and nonlocal scale coefficient using the ‘StandardScaler’ function from the scikit-learn preprocessing package [54]. This process is necessary to assure that all numerical features are in the same standard scale, facilitating model convergence and preventing certain features from being overshadowed by others. A dataset of 34,100 data points obtained by the scale-dependent nonlocal poroelastic model of small-scale spherical inclusions is used. The test size is set to 30%, making training and test datasets of 23,870 and 10,230 points, respectively. The ‘ColumnTransformer’ function from the scikit-learn compose package is utilised for the fast and robust transformation operation on the columns of the data frame with the inclusion’s features. A machine learning pipeline combined with a grid search cross validation framework is developed for an efficient and smooth hyperparameter tuning process. The scoring metrics for ranking machine learning models and finding the best configuration of hyperparameters is set to the negative root mean squared error. The number of estimators (decision tress) and leaves on each tree are taken in the range of 1–200 and 1–51, respectively, for the hyperparameter tuning. In addition, different values of learning rate between 0.01 and 0.2 and various maximum depths in the range of −1 to 100 are considered. Here, negative values are used to indicate that there is no restriction on the number of leaves. The machine learning pipeline includes three different boosting algorithms as GOSS, DART and GBDT. The best LGBM estimator with the minimum root mean squared error is obtained and applied for predicting the inclusion fluid pressure or radial displacement on unseen test data.

## 8. Results and Discussion

**Figure 3:**
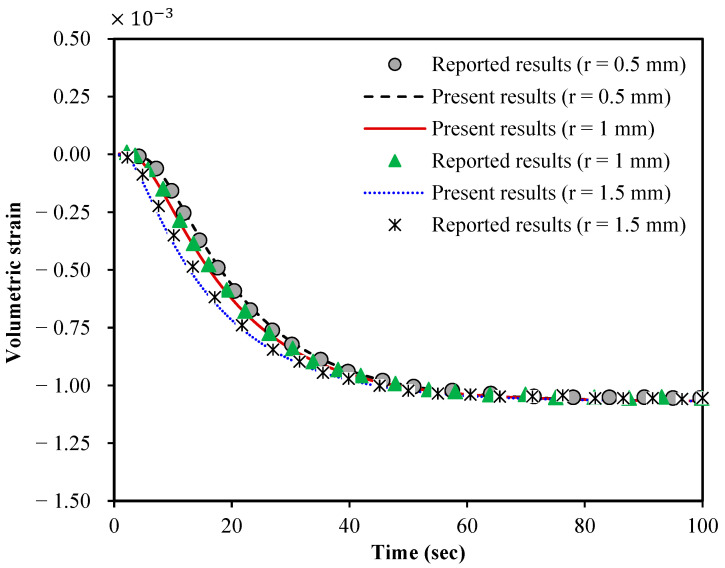
A validation study for the volumetric strain of large-scale local spherical tumours using classical poroelasticity; reported results are from Ref. [5]; the volumetric strain is defined as the sum of all normal strain components; the tumour radius is 3 mm, and the results are calculated at the two different locations *r =* 1 mm and 1.5 mm (*r* is measured from the tumour centre).

In this section, the results of the nonlocal poroelastic model and LGBM are presented and discussed on one of the most common applications of the inclusion–background models, which is the mechanical behaviour of solid tumours. First, to verify the accuracy of the nonlocal poroelasticity modelling, the volumetric strain of the present model is plotted in Figure 3 and compared to the one reported in Ref. [5] for local large-scale spherical tumours using the classical poroelasticity theory. The results are shown at various radial distances from the centre of the solid tumour. The tumour radius, Young’s modulus, Poisson’s ratio, hydraulic conductivity per volumetric weight and microfiltration coefficient are, respectively, taken as *R =* 3 mm, *E =* 97.02 kPa, v = 0.45, $\lambda_{hc}/\gamma_{vw}$ = 1.8 × 10^−13^ m^4^/Ns, $\chi_{tot}$ = 5 × 10^−9^ 1/Pa·s [5]. To make a reasonable comparison, scale effects related to the stress nonlocality are neglected. It is found that the results of our modelling approach closely match those reported in the literature.

**Figure 4:**
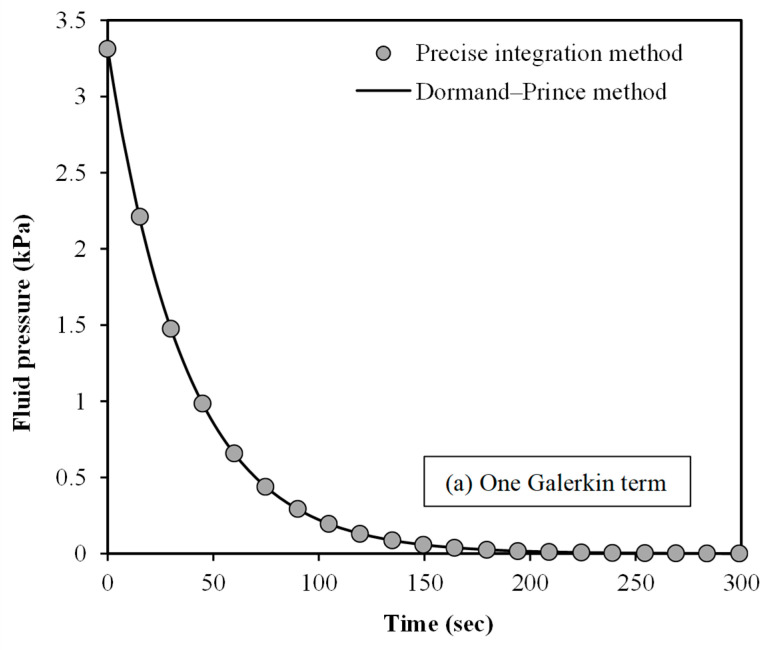

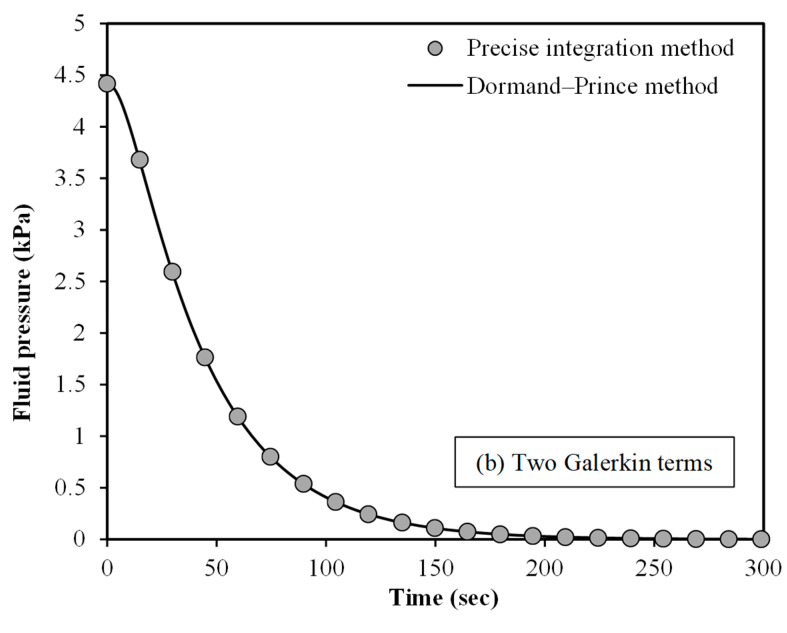
A validation study for the fluid pressure of small-scale spherical tumours for (**a**) one Galerkin term and (**b**) two Galerkin terms; size effects are incorporated using the scale-dependent nonlocal poroelasticity; the nonlocal scale coefficient is set to 0.2; the number of Galerkin terms refers to the number of base functions used to approximate the fluid pressure.

To further prove the validity of the mathematical scale-dependent modelling, the fluid pressure within the spherical tumour is plotted against time in Figure 4. The numerical results are demonstrated for two different solution procedures: (1) PIM and (2) Dormand–Prince method. Moreover, one and two Galerkin terms are assumed in Figure 4a,b, respectively. The fluid pressure is calculated at *r =* 0.5 *R*. The initial value of the dimensionless fluid pressure is set to 0.01. The Dormand–Prince solution procedure is implemented using a Matlab program. An excellent match is found between the two numerical techniques for the fluid pressure of spherical tumours using the scale-dependent nonlocal poroelasticity.

**Figure 5:**
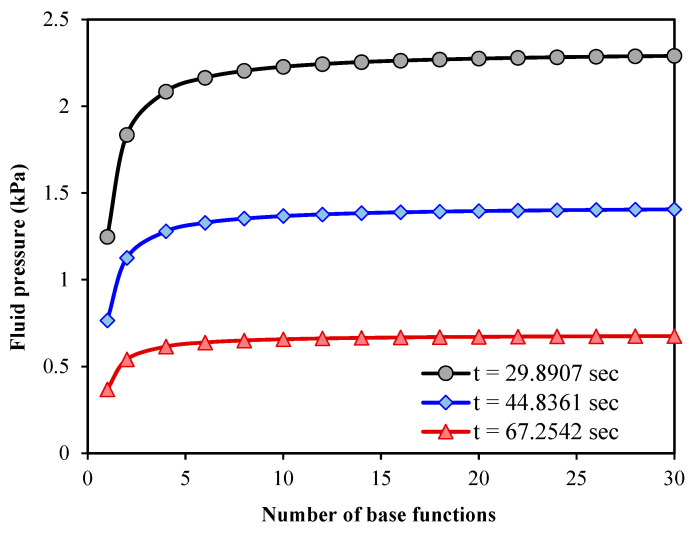
A convergence study for the solution procedure presented; 200 time steps are considered in numerical calculations using the PIM.

**Figure 6:**
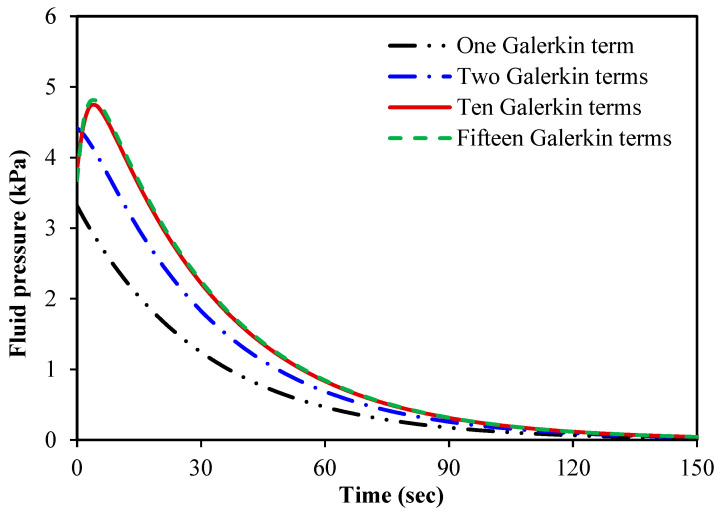
Fluid pressure of spherical tumours versus time for different Galerkin terms; the number of base functions of the radial displacement is the same as that of the fluid pressure.

To show the convergence of the solution, the tissue fluid pressure is shown in Figure 5 versus the number of base functions. The calculations are performed for three different time values. The tumour radius, Young’s modulus, Poisson’s ratio, hydraulic conductivity per volumetric weight and microfiltration coefficient are the same as those mentioned above for plotting Figure 3. The fluid pressure is numerically obtained at the midpoint between the tumour centre and surface. It is found that after about ten base functions, the results are converged in all cases. Figure 6 illustrates the fluid pressure of a spherical tumour against time for four various Galerkin terms (base functions). This figure shows how important it is to consider a sufficient number of Galerkin terms in calculating the fluid pressure of the tumour. Neither one nor two Galerkin terms are sufficient to obtain a reliable numerical solution. However, the cases of ten and fifteen base functions are very close to each other, which indicates that the results converge.

**Figure 7:**
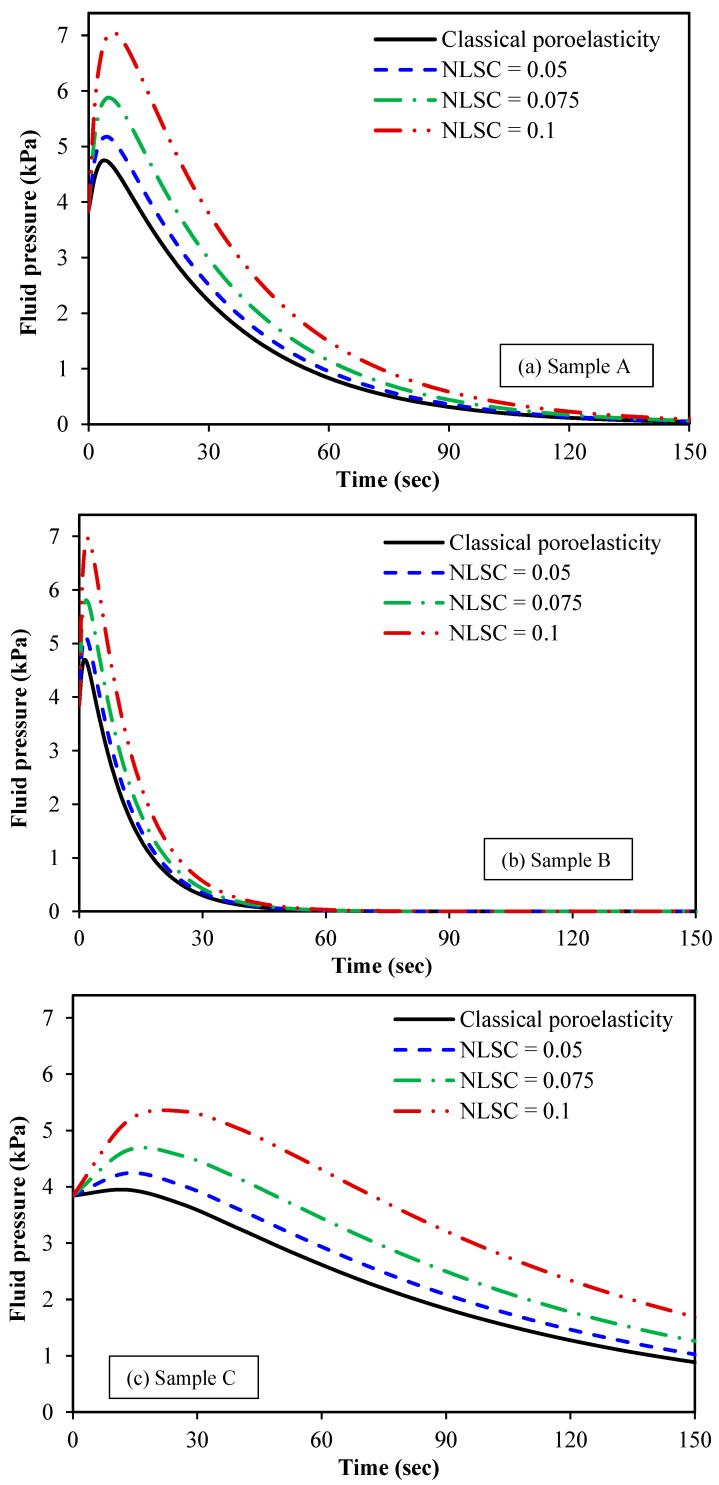
Fluid pressure of spherical tumours versus time for different nonlocal scale coefficients (NLSC): (**a**) sample A, (**b**) sample B and (**c**) sample C; the NLSC is defined as the ratio of the nonlocal parameter to the tumour radius; the nonlocal parameter is the product of the calibration parameter and the internal characteristics length; the average distance between two neighbouring cells inside the malignant tissue can be taken as the internal characteristic length of spherical tumours.

Figure 7 is plotted to discuss the influence of the nonlocal scale coefficient (NLSC) on the fluid pressure of the spherical tumour. The NLSC is defined as the ratio of nonlocal parameter to the tumour radius as $\gamma_{nl}=e_{0}a_{c}/R$, leading to a dimensionless scale parameter related to the stress nonlocality. Ten base functions are considered for both radial displacement and fluid pressure of the spherical tumour. Three different biological samples are taken into account for the spherical tumour. The poroelastic properties of these samples are listed in Table 1. The radius of the spherical tumour is set at *R =* ${3\times 10}^{3}\mu m$. For comparison purposes, the case of classical local poroelasticity, in which scale effects are ignored, is also considered. It is observed that as the NLSC is increased from 0 to 0.1, the fluid pressure at *r =* 0.5 *R* increases. This can be interpreted as one consequence of stiffness reduction due to the nonlocal effect. An increase in the NLSC leads to a considerable decrease in the structural stiffness of the tissue solid matrix, and this means that the tissue becomes softer and the pore fluid pressure is enhanced. From a clinical point of view, this finding is very important as it would result in improving the resolution of elastography imaging.

**Figure 8:**
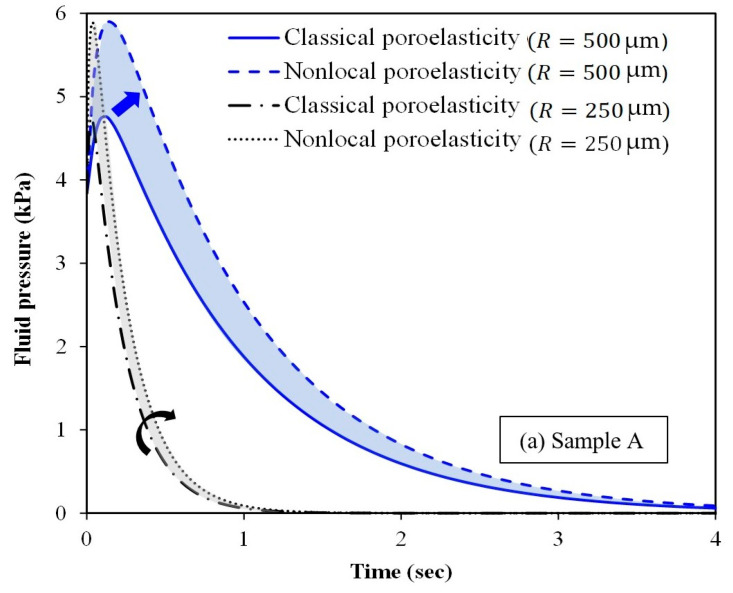

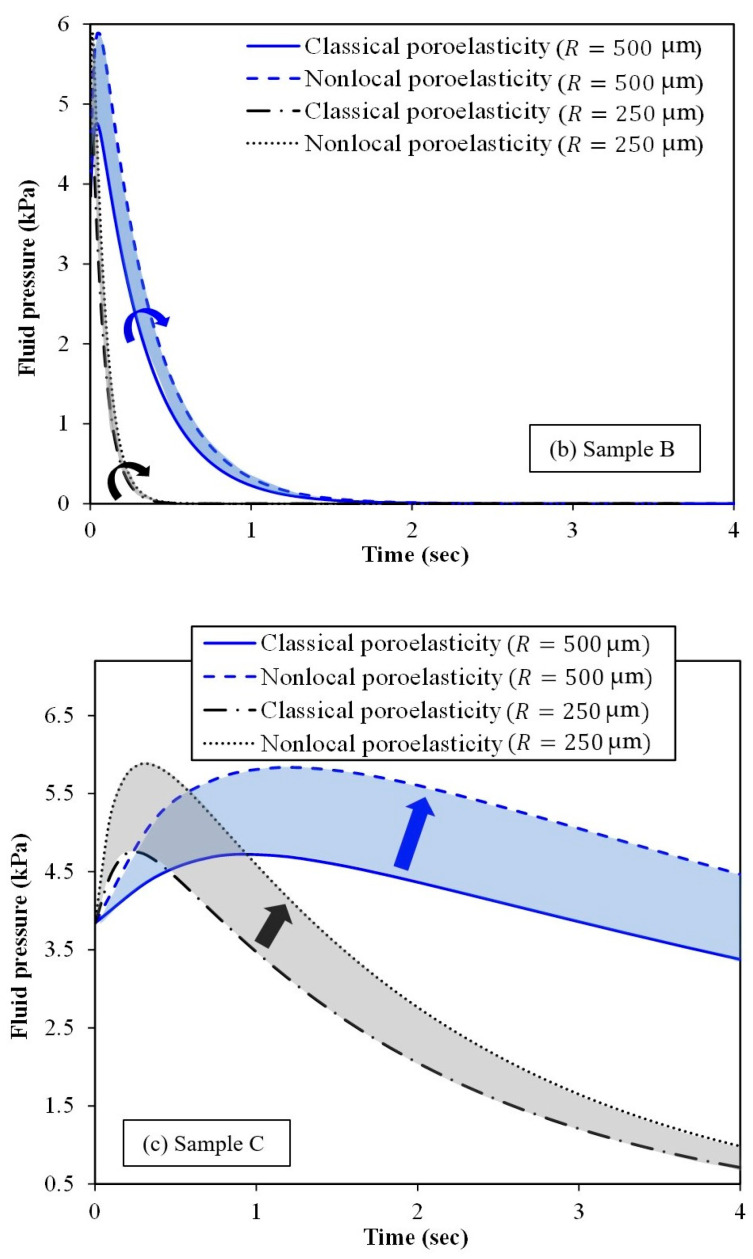
Fluid pressure of spherical tumours versus time for different tumour sizes: (**a**) sample A, (**b**) sample B and (**c**) sample C. Arrows indicate the improvement in the fluid pressure resolution by incorporating nonlocal effects.

Figure 8 depicts the effect of spherical tumour size and time on the fluid pressure at the half space between the tumour centre and surface. The figure also compares the nonlocal scale-dependent poroelasticity with the classical one for three different samples (samples A, B and C). When the radius of the spherical tumour decreases, the fluid pressure decreases as well. Furthermore, the fluid pressure gradually reduces over time. The only exception is the very early moments of imposing the applied compressive loading. At a certain time long enough after the loading, the fluid pressure vanishes inside the spherical tumour. For smaller tumours, the specific time corresponding to the loss of fluid pressure is considerably lower. Figure 8 demonstrates the promising capability of the nonlocal scale-dependent poroelasticity compared to the classical poroelasticity in estimating the fluid pressure within spherical tumours of ultrasmall sizes (less than 500 µm in radius). The clinical use of the proposed nonlocal poroelasticity model could result in a substantial improvement in the accuracy and sensitivity of the tissue mechanical property measurement using elastography imaging, especially for tumours of small-scale sizes.

**Figure 9:**
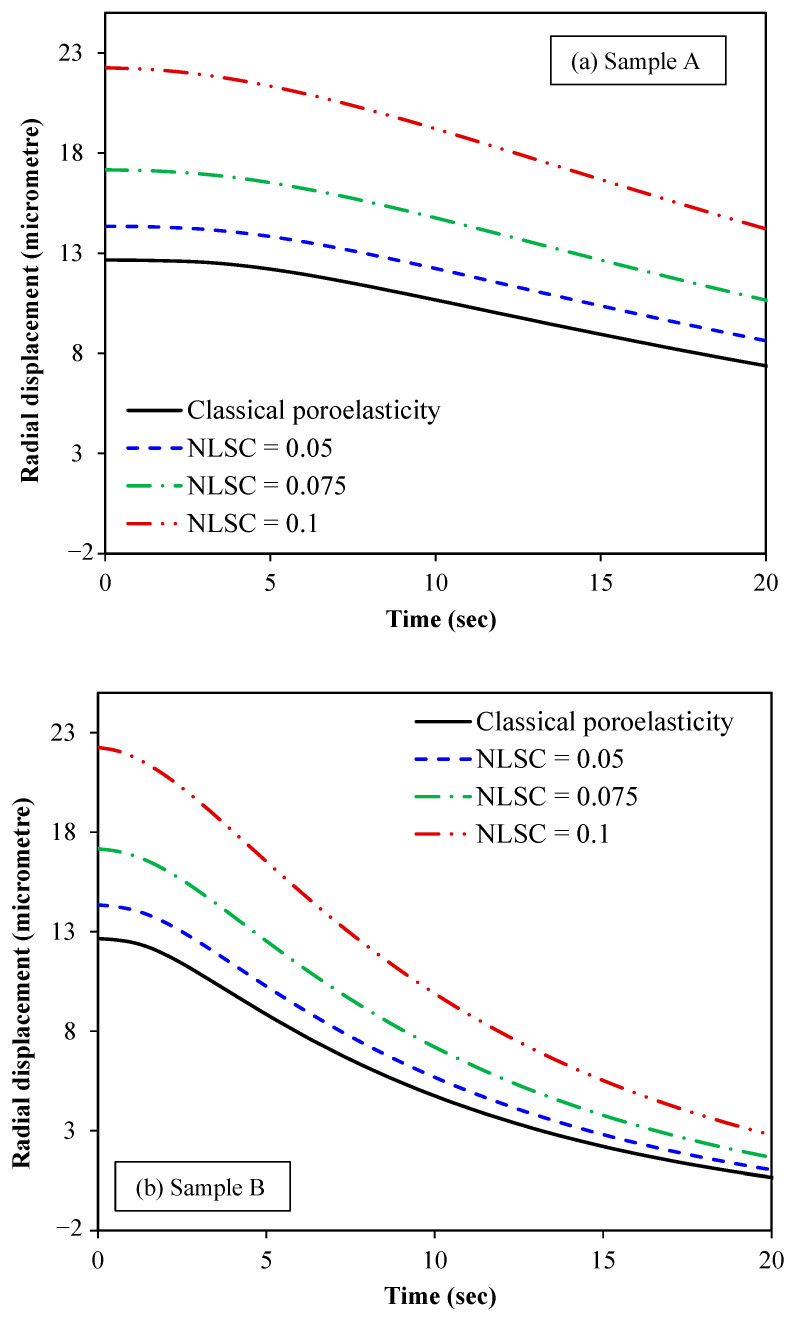

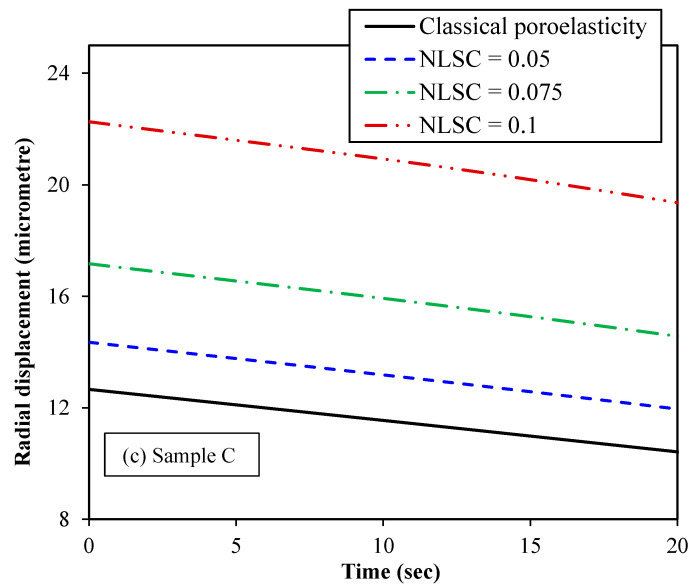
Radial displacement of spherical tumours versus time for different nonlocal scale coefficients (NLSC): (**a**) sample A, (**b**) sample B and (**c**) sample C. The classical poroelasticity model can be obtained from the nonlocal scale-dependent one when the effect of the NLSC is ignored. Ten base functions are considered for both the radial displacement and fluid pressure approximations. The radial displacement is obtained at *r =* 0.5 *R*.

The variation in the radial displacement with time is plotted in Figure 9 for various values of NLSCs for the three different biological samples. Young’s modulus, Poisson’s ratio and the geometrical features of the three samples are the same. However, they differ in terms of the hydraulic conductivity per volumetric weight and microfiltration coefficient, as can be seen from Table 1. The radial displacement is calculated at *r =* 0.5 *R*. Ten base functions (ten Galerkin terms) are supposed for both the radial displacement and fluid pressure of the spherical tumour in all case studies. From Figure 9, it can be concluded that the nonlocal scale coefficient has a vital role to play in the mechanical behaviour of small-scale tumours. As the scale effect related to the solid stress nonlocality inside the spherical tumour increases, larger radial displacements are observed. The validity of this finding is backed up by the evidence that nonlocal effects lead to a reduction in stiffness, making the tissue more prone to mechanical deformation. This finding is very important from a clinical point of view as the sensitivity and accuracy of the elastography-based cancer diagnosis could be significantly improved by taking into account these effects using the nonlocal poroelasticity theory.

A light gradient boosting machine (LGBM) algorithm is presented and integrated with the scale-dependent nonlocal poroelastic model of small-scale spherical inclusions. To show the accuracy and capability of the integrated model, the results of the nonlocal model for sample A are used as an example to build the training and test datasets. The radius of the inclusion, dimensionless nonlocal scale coefficient and time are used as the inputs of the LGBM model, while the fluid pressure at the middle distance from the inclusion centre to its surface is adopted as the label of the training and test datasets. Table 2 lists some general statistical information including the mean, median, maximum, minimum, first and third quartiles of the input features and fluid pressure as the target variable. The dataset includes 34,100 records, with 30% of them as the test data and 70% as the training data. A hyperparameter tuning procedure based on the grid search cross validation approach has been conducted to obtain the optimised parameters of the LGBM model. The negative root mean squared error is used to assess the performance of each configuration of the model parameters. The number of estimators and leaves on each tree are taken in the range of 1–200 and 1–51, respectively. Different learning rates between 0.01 and 0.2 and various maximum depths from −1 to 100 are also considered in the grid search cross validation. A negative maximum depth means that there is no limitation in terms of the number of leaves on the decision trees. Three different boosting types, GOSS, DART and GBDT, are considered in this analysis. Table 3 lists the results of the hyperparameter tuning for six different LGBM configurations. The mean test score is the negative root mean squared error of the training data. The optimised parameters of the best LGBM model are obtained as learning rate = 0.1, maximum depth = 100, number of decision trees = 200 and number of leaves = 51 (boosting type = GOSS). The root mean squared errors of this model on training and test data are 0.03389 and 0.03083, respectively. These values indicate the high accuracy of the LGBM model and no sign of overfitting as the performance of the model is even better on the unseen test data compared to the training data. In Table 4, the predicted fluid pressure is compared with the actual test fluid pressure obtained by the nonlocal model at the mid-distance from the centre to the surface of the spherical inclusion. Various values of the inclusion radii and nonlocal coefficients are taken into consideration. It is found that the results of the LGBM are in excellent agreement with those of the scale-dependent nonlocal model of poroelasticity, indicating the promising capability of the light gradient boosting frameworks to predict the mechanics of poroelastic inclusions.

**Figure 10:**
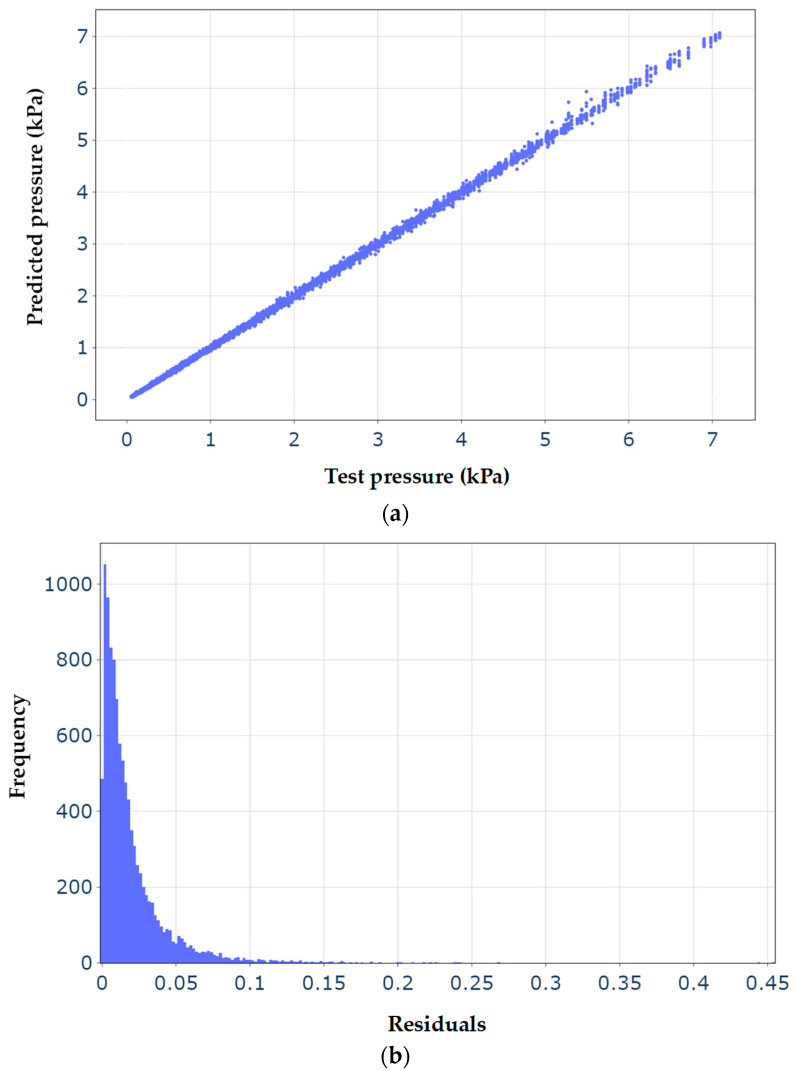
(**a**) Predicted pressure versus the actual test pressure in terms of kPa; the predicted pressure is obtained by the best light gradient bosting machine learning algorithm with the GOSS boosting type, learning rate = 0.1, maximum depth = 100, number of estimators = 200 and number of leaves = 51. (**b**) Histogram of the residuals of the fluid pressure for all data points of the test dataset; residuals are defined as the absolute value of “predicted pressure-test pressure”.

Figure 10a shows the variation in the fluid pressure of the small-scale spherical inclusion predicted by the best model of the LGBM versus the reference test fluid pressure obtained by the scale-dependent nonlocal poroelasticity model. To plot this figure, all 10,230 records of the test dataset are used to give an overview of the performance of the machine learning model. In addition, the histogram of the residuals of the fluid pressure within the spherical poroelastic inclusion is described in Figure 10b. The residuals are defined as the difference between the predicated and test fluid pressure. It can be concluded that the predicted fluid pressures closely match those of the test data almost in all cases. In addition, the majority of residuals are less than 0.075, providing an additional indicator of the goodness of the optimised LGBM model.

In the machine learning model, the fluid pressure at the middle of the inclusion radius is adopted to train the model and obtain optimised hyperparameters. The optimised model is then used to make reasonable estimations on unseen new data, as evidenced by our test data verification outlined above. In practice, there are two scenarios in which the present model would be useful: (1) When the size of the inclusion is determined by other imaging techniques such as magnetic resonance imaging (MRI) or computed tomography (CT); in this case, the model can be used to determine the inclusion type. For example, in biomedical applications, the model plays a crucial role in distinguishing benign tumours from malignant ones by comparing estimated mechanical characteristics with benchmark data. (2) A trial-and-error procedure for estimating the size of an inclusion involves systematically adjusting the parameters of a model until a satisfactory match is achieved between the predicted outcomes and observed data. In the context of this work’s case study on tumours and interstitial fluid pressure, this could refer to the process of iteratively refining the parameters related to the size of the tumour till the predictions align with experimental or clinical measurements. The present model relates the interstitial fluid pressure to the mechanical characteristics and size of the inclusion and could be useful in the iterative process to minimise the difference between the observation and theoretical estimation. In terms of proof of possibility, it is noteworthy that experimental studies have demonstrated that the interstitial fluid pressure is an important biomarker in solid tumours, significantly affecting the cancer microenvironment [55]. The clinical measurement of fluid pressure can be achieved using direct (invasive) techniques such as servo-controlled micropipette and wick-in-needle, as well as indirect (non-invasive) methods including ultrasound elastography and dynamic contrast MRI [56].

## 9. Conclusions

A nonlocal scale-dependent poroelasticity model has been developed for the mechanical response of spherical inclusions under radial compression. Scale effects related to the effective stress nonlocality were captured by using Eringen’s continuum mechanics. To derive the scale-dependent governing equations of the spherical inclusion, effective stress differential relations and the equilibrium equation were decoupled. The storage equation was derived based on the conservation of mass law for both fluid content and solid matrix. The Galerkin technique was employed to discretise the scale-dependent governing equation and the storage equation of the spherical inclusion, and then the numerical results were calculated using the PIM. For comparison and verification studies, a Dormand–Prince solution procedure and an analytical solution were presented for nonlocal small-scale and local large-scale inclusions, respectively. To obtain a reliable converged solution, ten base functions (Galerkin terms) were taken into consideration. The nonlocal model was integrated with an LGBM model for the fast and robust prediction of the mechanical behaviour of poroelastic inclusions in practical applications. The mechanical parameters calculated by the LGBM were in an excellent agreement with those estimated by the nonlocal continuum approach. It was found that nonlocal effects lead to a substantial increase in the fluid pressure within the spherical inclusion. Moreover, the radial displacement is underestimated using the classical local model of poroelasticity. These findings are rooted in the fact that the stress nonlocality is linked with a reduction in structural stiffness. Application of the proposed nonlocal scale-dependent poroelasticity model integrated with the LGBM results in a significant enhancement in the accuracy of the fluid pressure and radial displacement estimations within spherical inclusions subject to uniform radial loading. The specific time corresponding to the fluid pressure loss in the inclusion is greatly affected by the hydraulic conductivity per volumetric weight. The inclusions tend to consolidate much faster when the hydraulic conductivity is increased, and thus the specific time related to the fluid pressure loss is much lower. This leads to a constant radial displacement within the spherical inclusion, which is not dependent on time anymore.

## 10. Patents

Ali Farajpour, Wendy V. Ingman, “Scale-dependent elastography method for de-tection of small inclusions in biological tissue”. Applicant: The University of Adelaide, Application number: PCT/AU2023/050855. Patent Cooperation Treaty (PCT) Submission Date: 1 September 2023.

## Figures and Tables

**Table 1:** Poroelastic properties of three different tumour samples [5]; these properties were experimentally measured using mechanical testing. They were taken from the malignant lesions of human breast tissues. All three samples have the same Young’s modulus (*E*) and Poisson’s ratio (v); however, the hydraulic conductivity per volumetric weight and the microfiltration coefficient of the samples are different.

Sample Name	*E* (kPa)	v	$\lambda_{hc}/\gamma_{vw}\;(m^{4}/Ns)$	$\chi_{tot}$ (1/Pa·s)
A	97.02	0.45	1.80 × 10^−13^	5.00 × 10^−9^
B	97.02	0.45	5.103 × 10^−13^	5.67 × 10^−8^
C	97.02	0.45	2.04 × 10^−14^	5.67 × 10^−8^

**Table 2:** General statistical information about the training dataset used to fit the LGBM model. The first three columns include data about the average radius in micron, dimensionless nonlocal scale coefficient and time, which are considered as the inputs in the analysis. The fluid pressure column is considered as the target (label) column.

General Statistics	*R* (µm)	$e_{0}a_{c}/R$	*t* (s)	Fluid Pressure (kPa)
Count	34,100	34,100	34,100	34,100
Mean value	350	0.05	1.07270	1.45417
Standard deviation	89.44	0.03162	0.86654	1.64699
Minimum value	200	0	0	0.05269
First quartile	270	0.02	0.40255	0.21183
Median value	350	0.05	0.83377	0.69186
Third quartile	430	0.08	1.55312	2.22216
Maximum value	500	0.1	4.10998	7.08649

**Table 3:** Mean test scores of different boosting algorithms used in the LGBM model. The mean test score is used to rank the model in the hyperparameter tuning process. This scoring metric is set to the negative root mean squared error.

Boosting Type	Learning Rate	Maximum Depth	Number of Estimators	Number of Leaves	Mean Test Score
GOSS	0.1	100	200	51	−0.03389
GBDT	0.1	100	200	51	−0.03482
DART	0.2	no limit (−1)	200	21	−0.0752
GOSS	0.2	50	200	51	−0.03595
GBDT	0.1	no limit (−1)	170	51	−0.03626
DART	0.2	10	200	51	−0.06499

**Table 4:** Predicted pressure using the LGBM versus those of the test dataset for various values of inclusion radius, nonlocal scale coefficients and time. Percentage error is defined as “100 × abs(predicted pressure − test pressure)/test pressure” in which abs represents the absolute function.

*R* (µm)	$e_{0}a_{c}/R$	*t* (s)	Predicted Pressure (kPa)	Test Pressure (kPa)	Percentage Error (%)
210	0.09	0.2124	2.2592	2.3264	2.8886
300	0.08	0.7024	0.9083	0.9158	0.8190
350	0.09	1.5257	0.2832	0.2808	0.8547
470	0.07	1.9442	0.6444	0.6305	2.2046
270	0.02	0.2905	1.9325	1.9202	0.6406
440	0.04	3.1185	0.06314	0.0642	1.6511

## Data Availability

Data available upon request.
